# PD-L2 mediates tobacco smoking-induced recruitment of regulatory T cells via the RGMB/NFκB/CCL20 cascade

**DOI:** 10.1007/s10565-024-09892-3

**Published:** 2024-07-23

**Authors:** Hua Guo, Chen Zhang, Yu-Ke Shen, Jian-Dong Zhang, Fu-Ying Yang, Fan Liang, Wei Wang, Yu-Tao Liu, Gui-Zhen Wang, Guang-Biao Zhou

**Affiliations:** 1 State Key Laboratory of Molecular Oncology & Department of Medical Oncology, National Cancer Center/National Clinical Research Center for Cancer/Cancer Hospital, Chinese Academy of Medical Sciences and Peking Union Medical College, Beijing, 100021 China; 2 School of Life Sciences and Engineering, Handan University, Handan, Hebei Province 056005 China; 3 State Key Laboratory of Membrane Biology, Institute of Zoology, Chinese Academy of Sciences & University of Chinese Academy of Sciences, Beijing, 100101 China; 4 Department of Urology, Beijing Chaoyang Hospital, Capital Medical University, Beijing, 100020 China; 5 Shanxi Bethune Hospital Affiliated with Shanxi Academy of Medical Sciences, Taiyuan, Shanxi Province 030032 China; 6 School of Basic Medicine, Weifang Medical University, Shandong, 261000 China

**Keywords:** NSCLC, Tobacco smoke, PD-L2, RGMB, CCL20, Tregs

## Abstract

Programmed cell death ligand 2 (PD-L2), a ligand for the receptor programmed cell death 1 (PD-1), has an identity of 34% with its twin ligand PD-L1 and exhibits higher binding affinity with PD-1 than PD-L1. However, the role of PD-L2 in non-small cell lung cancer (NSCLC) progression, especially tobacco-induced cancer progression, has not been fully understood. Here, we found that PD-L2 promoted tumor growth in murine models with recruitment of regulatory T cells (Tregs). In patients with NSCLC, PD-L2 expression level in tumor samples was higher than in counterpart normal controls and was positively associated with patients’ response to anti-PD-1 treatment. Mechanismly, PD-L2 bound its receptor Repulsive guidance molecule B (RGMB) on cancer cells and activated extracellular signal-regulated kinase (Erk) and nuclear factor κB (NFκB), leading to increased production of chemokine CCL20, which recruited Tregs and contributed to NSCLC progression. Consistently, knockdown of RGMB or NFκB p65 inhibited PD-L2-induced CCL20 production, and silencing of PD-L2 repressed Treg recruitment by NSCLC cells. Furthermore, cigarette smoke and carcinogen benzo(a)pyrene (BaP) upregulated PD-L2 in lung epithelial cells via aryl hydrocarbon receptor (AhR)-mediated transcription activation, whose deficiency markedly suppressed BaP-induced PD-L2 upregulation. These results suggest that PD-L2 mediates tobacco-induced recruitment of Tregs via the RGMB/NFκB/CCL20 cascade, and targeting this pathway might have therapeutic potentials in NSCLC.

**Supplementary Information:**

The online version contains supplementary material available at 10.1007/s10565-024-09892-3.

## Introduction

Lung cancer is the primary cause of mortality worldwide. More than 90% of deaths from lung cancer are associated with tobacco smoking and air pollution, the two major public health concerns that have carcinogens including N-nitrosamines and polycyclic aromatic hydrocarbons (PAHs) ((US) et al. 2010; Huang et al. 2014; Mumford et al. 1987; Zhou 2019). Over the past decade, immune checkpoint inhibitors targeting programmed death-1 (PD-1) and programmed death-ligand 1 (PD-L1) have revolutionized the treatment of lung cancer (Antonia et al. 2018). Interestingly, Benzo[a]pyrene (BaP) is a representative PAH compound that has been reported to induce upregulation of PD-L1, conferring immune escape of cancer cells (Wang et al. 2019). BaP induces secretion of inflammatory factors (N’Diaye et al. 2006) and perturbs the immune system to facilitate cancer progression in cellular and animal models (Zhou 2019). However, the effects of BaP on modulating immunosuppressive cells and related mechanisms have not been fully understood.

PD-L2 is also a ligand for PD-1, has 34% of identity to PD-L1 and shares 20% homology with B7-1 (CD80) and B7-2 (CD86) (Lázár-Molnár et al. 2008). PD-L2’s binding affinity to PD-1 is two- to six-fold higher than that of PD-L1 (Ghiotto et al. 2010), and it competes with PD-L1 to bind PD-1 (Ghiotto et al. 2010) and inhibits the anti-tumor immunity through suppressing CD8^+^ T cells (Latchman et al. 2001). PD-L2 is highly expressed in head and neck squamous cell carcinoma (Yearley et al. 2017), renal cell carcinoma (Shin et al. 2016), pancreatic ductal adenocarcinoma (Zhang et al. 2019), and cervical cancer (2017). Meanwhile, PD-L2 is expressed in lung cancer (Fernandez et al. 2022), and high PD-L2 expression is associated with smoking, poor overall survival (OS) and disease-free survival (DFS) in patients with lung adenocarcinoma (LUAD) (Takamochi et al. 2022). However, the molecular mechanism of PD-L2 in lung cancer tumorigenesis, especially its immune-modulation requires further investigation.

Repulsive Guidance Molecule B (RGMB, also known as Dragon), a co-receptor for bone morphogenetic protein (BMP) signaling, is another receptor of PD-L2 (Xiao et al. 2014). RGMB binds to PD-L2, but not to PD-L1 (Xiao et al. 2014). In lung tissue, alveolar epithelial cells, interstitial macrophages, and dendritic cells may be involved in RGMB-PD-L2 interaction, whereas RGMB-deficient mice die 2-3 weeks after birth (Xia et al. 2011). RGMB renders initial T cell activation and expansion via directly binding to PD-L2, which promotes the development of respiratory immune tolerance (Nie et al. 2018; Xiao et al. 2014), while blockade of RGMB-PD-L2 interaction overcomes microbiome-dependent resistance to PD-1 pathway inhibitors (Park et al. 2023). Through binding to PD-L2, RGMB promotes colorectal cancer growth (Shi et al. 2015) and facilitates tumor metastasis and invasion in osteosarcoma (Ren et al. 2019; Sudo et al. 2020). On the contrary, reduced RGMB expression is associated with poor overall survival of patients with NSCLC (Li et al. 2016). These inconsistent results highlight the need to understand mechanisms by which the PD-L2/RGMB axis can regulate NSCLC tumor microenvironment and progression.

In this study, we aimed to evaluate the roles of PD-L2 in lung cancer pathogenesis, and found that this molecule promoted NSCLC progression *in vivo* with recruitment of regulatory T cells (Tregs). PD-L2 was inversely associated with clinical outcomes of the patients, especially in smoker patients. We found that BaP was able to upregulate PD-L2, which bound RGMB to activate NFκB and induced secretion of chemokine CCL20, leading to recruitment of Tregs to promote cancer progression. These results indicated the important role of PD-L2-RGMB-CCL20 in tobacco-induced lung carcinogenesis, and suggested that pharmacological modulation of this pathway may have therapeutic potentials in this deadly disease.

## Materials and methods

### Patients and tissue specimens

This study was approved by the research ethics committees of Cancer Hospital, Chinese Academy of Medical Sciences and Peking Union Medical College (NCC2020A190), Sun Yat-Sen University Cancer Center and Shanxi Bethune Hospital affiliated with Shanxi Academy of Medical Sciences. All lung cancer samples were collected with informed consent. Patients admitted to the hospital with a diagnosis of lung cancer by at least two independent pathologists were included in this study. Samples with fresh normal lung tissues (5 cm away from tumor lesions) were obtained from surgical specimens resected during the surgery and immediately frozen in liquid nitrogen for further analysis. A total of 45 frozen samples were obtained to detect the expression of PD-L2 in human lung tumor specimens and the adjacent normal lung tissues, while 25 formalin-fixed and paraffin embedded (FFPE) specimens according to manufacturer’s protocol were collected from lung cancer patients receiving nivolumab immunotherapy to determine the correlation of PD-L2 and FOXP3 expression levels.

### Cell lines and cell culture

Human lung cancer cell lines (H1299, H1975, H460, H520, H446, and H82), murine cancer cell lines (LLC, MC-38, and Ag104Ld), and human embryonic kidney line (HEK293T) were purchased from the American Tissue Culture Collection (ATCC; Manassas, VA, USA), while the human normal bronchial epithelial cell line 16HBE was obtained from Lonza Clonetics (Clonetics, Walkersville, MD, USA), and human embryonic lung fibroblast cell line HLF was purchased from Kenqiang Instrument Co., Ltd (Shanghai, China). These cells were cultured in Dulbecco Modified Eagle Medium (DMEM) or Roswell Park Memorial Institute (RPMI) 1640 supplemented with 10% fetal bovine serum (FBS, Gibco, Grand Island, NY, USA), 100 U/mL penicillin, and 100 μg/mL streptomycin in a humidified incubator at 37°C with 5% CO_2_.

### Antibodies and reagents

The antibodies used in this study were as follows: rabbit anti-human PD-L2 (#82723, Cell Signaling Technology, Beverly, MA, USA; 1:100 for immunohistochemistry (IHC)), rabbit anti-human PD-L1 (#13684, Cell Signaling Technology, Beverly, MA, USA; 1:100 for IHC), rabbit anti-human FOXP3 (#98377, Cell Signaling Technology, Beverly, MA, USA; 1:100 for IHC), anti-p-p65 (#3033, Cell Signaling Technology, Beverly, MA, USA; 1:1000 for western blot), anti-PD-L2 (#ab87662, Abcam, Cambridge, MA, USA; 1:1000 for western blot), mouse anti-PD-L2 (#bsm-30107M, Bioss antibodies, Beijing, China), rabbit anti-RGMB (#bs-11474R, Bioss antibodies, Beijing, China; 1:1000 for western blot), anti-RGMB (#A12859, Abclonal, China; 1:1000 for western blot), anti-PD-L2 (#ab288298, Abcam, Cambridge, MA, USA; 1:50 for Co-immunoprecipitation); anti-β-Actin (#A1978, Sigma, St. Louis, MO, USA; 1:5000 for western blot), anti-PD-L2 (#sc-57398, Santa cruz, USA; 1:50 for IHC), anti-PD-L2 (#NBP1-76770, Novus iologicals, LLC, USA; 1:100 for immunofluorescence assay), anti-p65 (#A19653, Abclonal, China; 1:1000 for western blot), anti-AhR (#83200, Cell Signaling Technology, Beverly, MA, USA; 1:1000 for western blot, 1:50 for Chromatin immunoprecipitation (CHIP)), Zombie Aqua™ dye (#423101,Biolegend; 1:1000 for flow cytometry), BUV395 anti-mouse CD45 (#745698, BD OptiBuild, 1:100 for flow cytometry), APC anti-mouse CD4 (#100411,Biolegend; 1:20 for flow cytometry), PE/Cy7 anti-mouse CD8a (#100721,Biolegend; 1:20 for flow cytometry), APC/Cy7 anti-mouse CD11b (#101225, Biolegend; 1:20 for flow cytometry), PE/Cy5 anti-mouse F4/80 (#123111, Biolegend; 1:20 for flow cytometry), PE anti-mouse Gr1 (#108407, Biolegend; 1:20 for flow cytometry), APC anti-mouse CD206 (#141707, Biolegend; 1:20 for flow cytometry), PE/Cy7 anti-mouse MHCII (#107629, Biolegend; 1:20 for flow cytometry), PE/Cy7 anti-mouse CD86 (#105013, Biolegend; 1:20 for flow cytometry), PE anti-mouse FOXP3 (#12-4776-41, eBioscience; 1:20 for flow cytometry), PE anti-human PD-L1 (#329705, Biolegend; 1:20 for flow cytometry), and PE anti-human PD-L2 (#329605, Biolegend; 1:20 for flow cytometry). Benzo(a)pyrene (#B1760), Alpha-Naphthoflavone (ANF; #N5757), Puromycin, and RNase A were purchased from Sigma-Aldrich. PD98059 was purchased from Selleck Chemicals.

### Flow cytometry analysis

The 16HBE, HLF, H446, H82, H1975, H460, H520, LLC, MC-38, and Ag104Ld cells with or without IFN-γ treatment were collected and incubated with PE-conjugated anti-PD-L2 (#329605, Biolegend) or PE-conjugated anti-PD-L1 (#329705, Biolegend) on ice. The cells incubated with isotype control was set as negative control. After 30 min incubation, the cells were washed twice with PBS, and subjected for flow cytometry analysis by using BD Fortessa 421 (LSRFortessa X-20, BD, USA).

### RNA extraction and quantitative real-time PCR (qRT-PCR)

Total RNA was extracted from cells using the Trizol Reagent (Invitrogen, Frederick, MD, USA), and the concentration of isolated RNA was measured by using a NanoDrop spectrophotometer 2000 (Thermo Fisher Scientific). Then 2 μg of RNA was reverse-transcribed into cDNA using a 1st-STRAND cDNA Synthesis Kit (Fermentas, Pittsburgh, PA, USA) in accordance with the manufacturer’s protocol. Follow the transcription, qRT-PCR was performed using SYBR PremixExTaq (Takara Biotechnology, Dalian, China). The sequences of the primers used were listed in Supplementary Table 1. GAPDH was used as an internal reference gene, and the relative expression of genes was calculated using the 2^−ΔΔCt^ method. The sequences of siRNAs, shRNAs, and sgRNAs were listed in Supplementary Table 1.

### Immunohistochemistry analysis

Briefly, formalin-fixed, paraffin-embedded human or mouse lung cancer tissue specimens (5 μm) were deparaffinized using xylene and graded alcohol, and then subjected to a heat-induced epitope retrieval step in citrate buffer solution. Then sections were blocked with 5% BSA for 30 min, and incubated with indicated primary antibodies at 4°C overnight. After that, sections were incubated with secondary antibodies for 90 min at 37°C, and then stained with 3, 30-diaminobenzidine (DAB, Zhongshan Golden Bridge Biotechnology, Beijing, China). Three fields of view per sample were imaged and analyzed. Immunoreactivity scores (IRS) were calculated by IRS (0–12) = RP (0–4) × SI (0–3), where RP is the percentage of positive cells and SI is staining intensity. For the multiplex immunohistochemistry assay, the PANO 4-plex IHC kit (PN130721AD, Panovue, Beijing, China) was used according to the manufacturer’s instructions. Specimens were incubated with anti-FOXP3 (#98377, Cell Signaling Technology) and anti-PD-L2 (#82723, Cell Signaling Technology). Slides were scanned and analyzed using the PerkinElmer Mantra Quantitative Pathology Imaging System (Waltham, Massachusetts, USA).

### Immunofluorescence microscopy

Cells were plated on the coverslips (24 mm x 24 mm) and then fixed with 4% paraformaldehyde for 15 min. After permeabilized with 0.3% Triton X-100 in PBS for 20 min and blocked with 5% BSA, the cells were incubated with indicated primary antibodies overnight at 4°C. Then the cells were subjected to PE-labeled secondary antibody or Alexa Flour® 488/647-labeled secondary antibody (Life technologies) in PBS for 2 h. The resulting cells were imaged using a laser scanning confocal microscope (Zeiss, Oberkochen, Germany).

### Lentivirus-mediated transfection

To generate PD-L2 stable knockdown or overexpression cells, the shRNA sequences targeting human *PD-L2* were inserted into a PLKO.1 vector to construct the PLKO.1/sh*PD-L2* expression vector, while the cDNA sequences of *PD-L2* were subcloned into pCDH-GFP vector to construct the pCDH-GFP/*PD-L2* expression vector. Then these vectors were co-transfected with psPAX2 and pMD2G into HEK293T cells. After 6 h transfection, the culture medium was replaced with fresh medium, and the lentiviral vector-containing supernatants were harvested 48 h and 72 h post transfection and centrifuged to obtain lentiviral particles. Targeted cells were infected with lentiviral particles in the presence of 8 μg/mL polybrene, and expression of GFP was used as a quantitative measure of infection using a MoFlo XDP cell sorter (Beckman Coulter).

### Enzyme-linked immunosorbent assay (ELISA) and Co-immunoprecipitation (Co-IP)

Cells were plated in the 6-well plates at a density of 1x10^5^ cells per well and incubated at 37°C for 48 h. Then the supernatants from each well were collected, and the CCL20 concentration in culture supernatants of lung cancer cell lines was measured by ELISA kit (#441404, Biolegend, USA) as previously described (Lian et al. 2021). The samples were diluted 2-fold before CCL20 ELISA assay analysis. For Co-IP, the PD-L2-overexpressed cells were cultured in a 100 mm-culture dish and lysed in immunoprecipitation lysis buffer containing phosphatase inhibitors and protease inhibitors. The cell lysate supernatant was obtained via centrifugation at 4°C (12,000 g, 15 min), and then incubated with protein A/G-linked magnetic beads (Santa Cruz, CA, USA) and anti-PD-L2 antibody at 4°C overnight. The immunoprecipitants were washed 5 times with immunoprecipitation lysis buffer and boiled with 2xSDS loading buffer. After that, the Co-IP samples were subjected to western blot analysis.

### Proximity ligation assay (PLA)

H1975 and H460 cells were seeded at a 6-well plate and transfected with exogenous PD-L2 plasmid. After 24 h of transfection, cells were fixed in 4% paraformaldehyde for 30 min and washed with PBS. The proximity of PD-L2 to RGMB was assessed using the Duolink® In Situ PLA® Probe Anti-Rabbit PLUS, Duolink® In Situ PLA® Probe Anti-Mouse MINUS, and Duolink® PLA In Situ Detection Reagents Red (Sigma-Aldrich, St. Louis, MO, USA) according to the manufacturer’s instructions. Specifically, the rabbit anti-RGMB antibody (1:200) and mouse anti-PD-L2 antibody (1:200) were used for PLA assay. Fluorescent images were taken using a laser scanning confocal microscope.

### Isolation of lymphocytes

Human peripheral blood mononuclear cells (PBMCs) were isolated from the heparinized peripheral blood of healthy volunteers by Ficoll density gradient-based separation. Then human CD4 Microbeads (Miltenyi Biotec, catalog 130-045-101) were used to isolate CD4^+^ T cells from the PBMCs according to the manufacturer’s instructions. CD4^+^CD25^+^ Tregs were further sorted using flow sorting.

### Transwell migration assays

Transwell migration assays were carried out in 24-well plates with inserts (5 μm, Corning Incorporated, Corning, NY, USA) according to the manufacturer’s instructions. Briefly, Tregs (1 × 10^5^ cells/well) or PBMCs (2 × 10^5^ cells/well) were plated to the upper chamber, and 600 μL RPMI-1640 medium containing recombinant human CCL20 (10 ng/ml; Peprotech, USA), anti-CCL20 antibody (1 ng/ml; AF360-SP, R&D, USA), supernatants taken at 24 h from pure cultures of *PD-L2*-overexpressed cells or sh*PD-L2* transfected cells were added to the lower chambers. After 24 h incubation, cells migrated to the lower chamber were manually counted.

### Western blot analysis

The cells were lysed in RIPA lysis buffer (50 mM Tris-HCl (pH 7.5), 150mM NaCl, 1mM EDTA, 1 mM MgCl_2_, 0.5% Triton X-100) supplemented with phosphatase inhibitors and protease inhibitors. Protein concentration was determined by using the BCA method (Biyuntian, China). Equal amount of protein (50 μg) from each sample was loaded and separated on a 10% SDS-PAGE gel, and then transferred to nitrocellulose blotting membranes (GE Healthcare Life science). After blocking with 5% non-fat milk in Tris-buffered saline containing 0.1% Tween-20 (TBST), membranes were incubated with the indicated primary antibodies overnight at 4°C. Then membranes were washed with TBST and incubated with the corresponding secondary antibodies at room temperature. GAPDH and β-actin were used as internal controls. The immunocomplex on the membrane was visualized using Luminescent Image Analyzer LSA 4000 (GE, Fairfield, CO, USA).

### Luciferase-based reporter assay

The H460 cells were seeded in 12 well plates and incubated overnight for adhesion. After reaching 60% confluence, cells were co-transfected with Firefly luciferase plasmid under the control of wild-type or mutant PD-L2 promoter and Renilla luciferase plasmid for 6 h. Then the medium was refreshed and cells were treated with BaP for additional 48 h. After that, cells were lysed in lysis buffer for 15 min, and the supernatant was collected for luciferase activity measurement. Firefly and Renilla luciferase activities were measured by a dual-luciferase reporter assay system (Promega, Madison, WI) according to the manufacturer’s instructions. The PD-L2 promoter activity was expressed as ratio of Firefly luciferase to Renilla luciferase.

### Chromatin immunoprecipitation (ChIP) assay

The H460 cells were treated or untreated with 5 μM BaP for 48 h, collected and fixed with 1% formaldehyde. Then the cell pellets were lysed in RIPA lysis buffer and sheared using sonication. The specific protein/DNA complexes were immunoprecipitated using an antibody against AhR (#83200, Cell Signaling Technology, 1:50). Following immunoprecipitation, cross-linking was reversed, the residual RNA was digested by RNase A (10 μg/mL), while the proteins were removed by proteinase K (40 μg/mL). The resulting DNA was purified and analyzed by quantitative PCR (qPCR). The primers used to detect XRE1 were listed in Supplementary Table 1.

### CRISPR/Cas9 assay

The CRISPR/Cas9 assay was performed as previously described by Feng Zhang, et al (Ran et al. 2013). Briefly, the lentiCRISPR plasmid pX330 obtained from Addgene was digested by *BsmB*1. The pX330-sgAhR plasmids expressing human Cas9 and sgAhR were prepared by ligating sgAhR oligonucleotides into the *BsmB*1 site of pX330. The sgRNA target sequence for AhR was listed in Supplementary Table 1. Then the H520 cells were transfected with constructed plasmids for 48 h. The single positive cells were sorted by fluorescence activated cell sorting (FACS) into 96-well plates. After three weeks after single-cell sorting, cells were collected to determine the knock-out efficacy by sequencing and western blot analyses.

### Animal study

The animal studies were approved by the Institutional Review Board of Institute of Zoology, Chinese Academy of Sciences, and were conducted according to protocols approved by the Animal Ethics Committee of the Institute of Zoology, Chinese Academy of Sciences. Female C57BL/6 mice (5-6 weeks old) were purchased from the Vital River Laboratory Animal Technology Co. Ltd. (Beijing, China). Female A/J mice (5-6 weeks old) and homozygous AhR-deficient mice were purchased from the Jackson Laboratory (Bar Harbor, Maine, USA). The A/J mice were exposed to cigarette smoke or treated with BaP to develop lung tumors as previously described (Wang et al. 2019). Briefly. The A/J mice (*n* = 7 per group) were exposed to cigarette smoke generated by DSI’s Buxco Smoke Generator (Buxco, NC, USA) at a frequency of 12 cigarettes per day, 5 days per week for 60 days. The duration of cigarette smoke exposure per cigarette was 3 minutes, followed by a 15-minute fresh air exposure. In other settings, the A/J mice (*n* = 7 per group) were orally administrated with 100 mg/kg of BaP twice a week for 5 weeks. The A/J mice were sacrificed on the 60^th^ day after administration for further experiments. The 60-day duration was to make the likelihood of lung tumorigenesis in mice higher (Wang et al. 2019). The C57BL/6 mice were injected with LLC cells (5 ×10^5^) via tail vein to establish lung cancer mouse model or subcutaneously implanted with 5 ×10^5^ of MC-38 cells. Tumor growth was monitored by micro-CT (Quantum FX, PerkinElmer, USA) for LLC mouse model or measured using a digital Vernier caliper for MC-38 mouse model. The tumor volume for MC-38 mouse model was calculated by the formula: volume (mm^3^) = (width)^2^ × length /2. At the end of the experiment, all animals were sacrificed with CO_2_ overdose before cervical dislocations and tumor or lung tissues were collected, weighted, and stored for further experiments.

### *Ex vivo* flow cytometry analysis

The mouse lung tissues were dissected into 2 mm pieces, and digested by collagenase IV (0.3%; Sigma) at 37 °C for 1 h. A single-cell suspension was obtained through a 70 μm cell strainer (BD Falcon, BD Biosciences, USA). The cells were then centrifugated and resuspended in ACK lysis buffer (Beyotime Biotechnology, China) for erythrocyte removal. After that, the resulting cells were labeled with indicated antibodies (BioLegend) and sorted by BD Fortessa 421. The data were analyzed on FlowJo (BD). The Zombie Aqua™ dye was used to determine live cells. The Foxp3^+^ cells gating on live CD45^+^CD3^+^CD4^+^ T cells represented Treg cell, the MHCII^+^ cells gating on live CD45^+^Gr1^+^ cells represented MDSC cell, the CD86^+^ cells gating on CD45^+^CD11b^+^F4/80^+^ cells represented M1 cell, and the CD206^+^ cells gating on live CD45^+^CD11b^+^F4/80^+^ cells represented TAM cell. Matched isotype controls were used in all experiments.

### Statistical analyses

All experiments were carried out at least in triplicate. The statistical analyses were performed using Graph Pad Prism 8 software (Graph Pad Software, La Jolla, CA, USA). Data obtained from different groups were compared using Student’s *t* test, chi-squared test, or one-way analysis of variance (ANOVA). The log-rank test was applied for survival analysis. Spearman correlation analysis was performed to determine the relationship between variables. Fisher exact test was used for the correlation comparison of characteristics of the NSCLC patient. *P* < 0.05 was considered statistically significant.

## Results

### PD-L2 promotes lung cancer progression with an increase in Treg infiltration

**Fig. 1:**
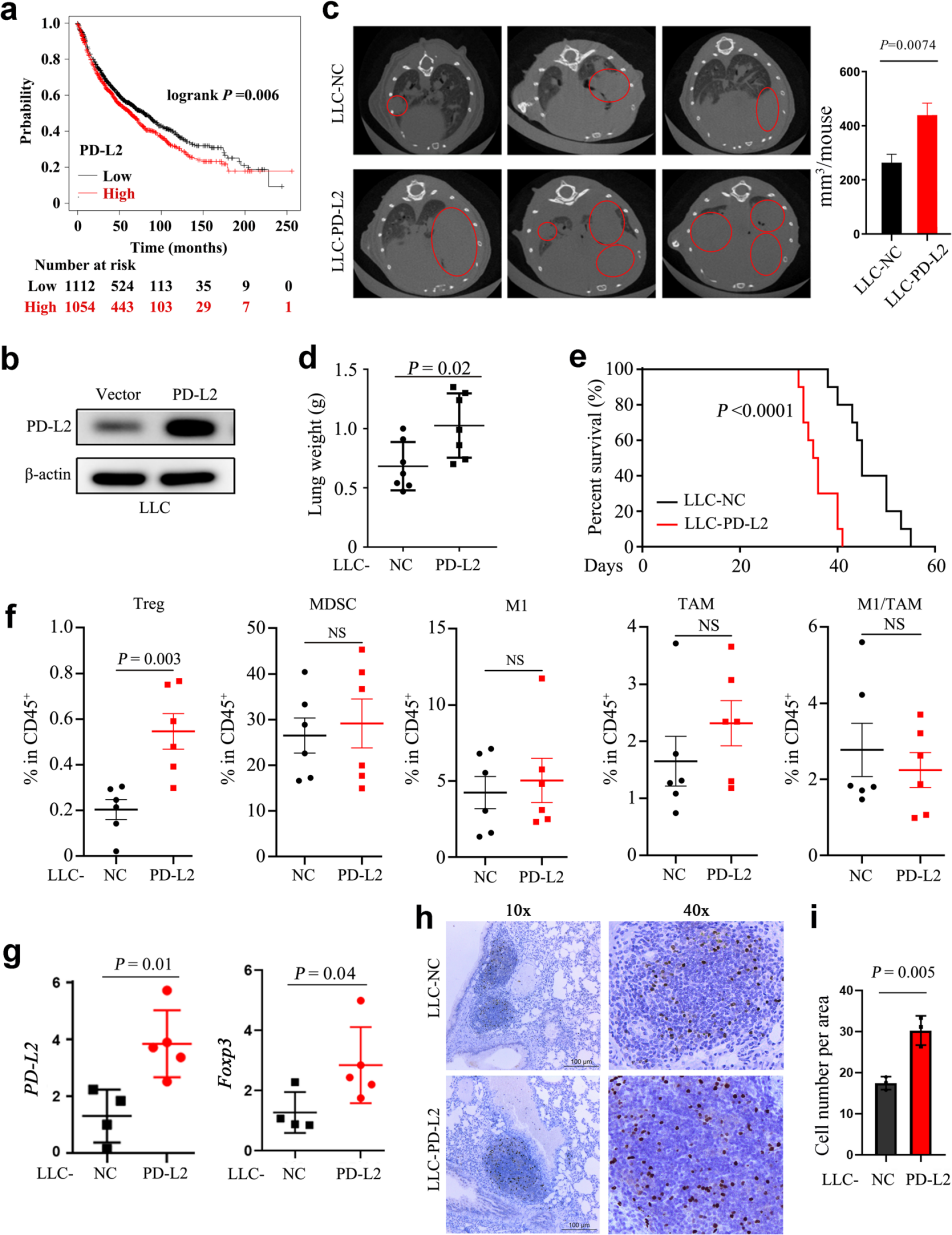
PD-L2 promotes lung cancer progression with recruitment of Tregs. **a** Kaplan-Meier Plotter database was used to assess the effects of *PD-L2* prognostic value in NSCLC patients. **b** Construction of *PD-L2* stably overexpressed LLC cell line. The PD-L2 expression was examined by western blot. **c** Micro-CT scanning of tumor burden in lung tissues of mice 28 days after intravenous injection of LLC-NC or LLC-*PD-L2* cells. The representative micro-CT images (*n* = 3 per group) were shown. Red circles in the micro-CT images represent tumor nodes in the lungs, and the quantitative results of tumor burden in these two groups were shown. **d** Wet lung tissue weights in LLC tumor-bearing mice (*n* = 7 per group). **e** The overall survival of mice after LLC-NC or LLC-*PD-L2* cell intravenous injection (*n* = 10 per group). The log-rank test was applied for survival analysis. **f** Flow cytometry analysis of the percentage of Tregs, MDSC, M1, TAM, and M1/TAM in the LLC-NC or LLC-*PD-L2* tumors (*n *= 6 per group). **g** The mRNA expression level of *PD-L2* and *FOXP3* in lung tissues of the mice in LLC-NC (*n *= 4) or LLC-*PD-L2* (*n *= 5) groups. **h** The immumohistochemistry staining of FOXP3^+^ Treg cells in lung tissues of mice (*n *= 3 per group) inoculated with LLC-NC or LLC-*PD-L2* cells. **i** Quantification of FOXP3^+^ Treg cells in tumor nodes of lung tissues. Treg, regulatory T cells; MDSC, myeloid-derived suppressor cells; M1, macrophage; TAM, tumor-associated macrophage. Student’s *t* test was used unless noted otherwise, *P < 0.05; **P < 0.01. Error bars, sem

We first tested the potential association between *PD-L2* expression level and clinical outcome of NSCLCs using the Online Survival Analysis Software (Gyorffy 2024), and found that the expression level of *PD-L2* was inversely associated with overall survival (OS) in lung cancer patients (Fig. 1a, Supplementary Fig. 1), though the expression level of *RGMB* was not associated with the clinical prognosis of NSCLC patients (Supplementary Fig. 2).

We tested PD-L2 expression in a few human and murine cancer cell lines by flow cytometry, and found that PD-L2 was expressed in human lung cancer cell lines (H1975, H460, and H520) and bronchial epithelial cell line (16HBE) (Supplementary Fig. 3). Notably, the PD-L2 expression in the three murine lung cancer cell lines was relatively low (Supplementary Fig. 3). Therefore, we constructed murine Lewis lung carcinoma (LLC) cell line that stably overexpressed PD-L2 to evaluate its effect on lung tumorigenesis in mice (Fig. 1b). Negative control (NC)-expressing LLC (LLC-NC) and PD-L2-expressing LLC (LLC-PD-L2) cells were intravenously injected into mice to generate lung cancer mouse model, and tumor growth was monitored by micro-CT imaging. The results showed that lung tumor burden in mice bearing LLC-PD-L2 xenografts were greater than that in control group (Fig. 1c). The lung tissues were harvested and weighted. As shown in Fig. 1d, lung weight in LLC-PD-L2 group was significantly increased than that in control group (*P* < 0.05), suggesting the greater tumor burden in the mice. Overexpression of PD-L2 in LLC cells significantly shortened survival time of mice bearing xenograft tumors (*P* < 0.001, Fig. 1e). We also constructed murine colon cancer (MC-38) cell line that stably overexpressed PD-L2 (Supplementary Fig. 4a), and the effects of PD-L2 on tumor growth were assessed in MC-38-xenograft model. Similarly, the tumor growth was significantly enhanced in mice subcutaneously injected with MC-38-PD-L2 cells than MC-38-NC cells (*P* < 0.05, Supplementary Fig. 4b-d). Tumor-infiltrating suppressive immune cells were dissociated from LLC-NC and LLC-PD-L2 tumors and analyzed by flow cytometry. Results showed that the percentage of Tregs in LLC-PD-L2 tumors was significantly higher than in control tumors (*P* < 0.01, Fig. 1f, Supplementary Fig. 5), while there was no significant difference in myeloid-derived suppressor cells (MDSC), macrophage (M1), tumor-associated macrophage (TAM), and M1/TAM ratio (Fig. 1f) between two groups.

Tregs could suppress many antitumor immune cell subsets to promote tumor growth, while FOXP3 is reported a master regulator in the development and function of Tregs. We analyzed the mRNA expression level of *FOXP3* in lung tissues of the mice of the two groups, and found that *FOXP3* as well as *PD-L2* expression levels were elevated in tumor tissues of LLC-PD-L2-bearing mice than that in LLC-NC-bearing mice (Fig. 1g). The immumohistochemistry staining of murine lung tissues showed that more FOXP3^+^ Treg cells were present in LLC-PD-L2 group than that in control group (Fig. 1h-i).

### The expression level of PD-L2 is correlated with FOXP3

**Fig. 2:**
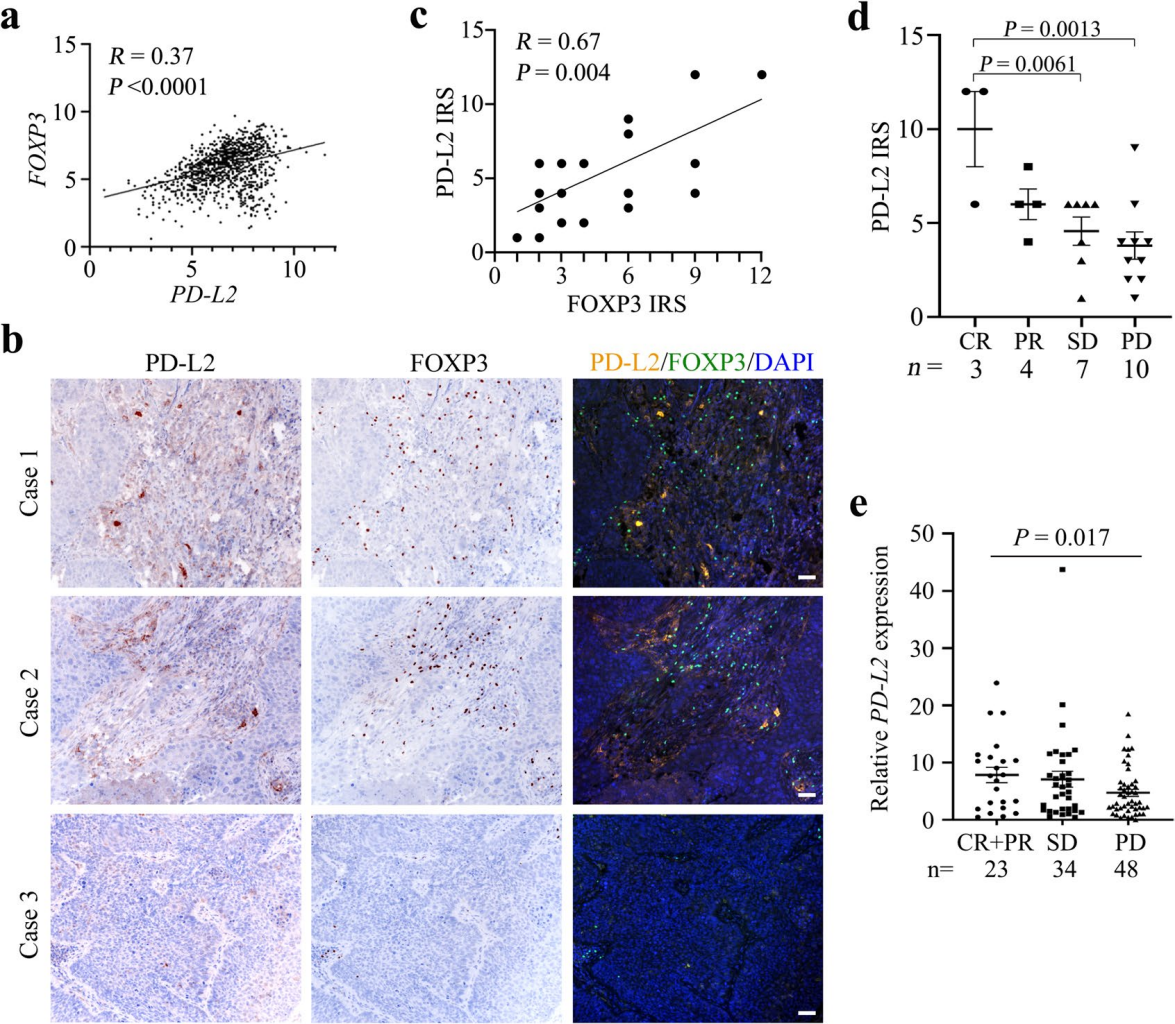
The expression level of PD-L2 is associated with FOXP3 and clinical outcomes of patients treated with anti-PD-1 antibodies. **a** The correlation between the expression level of *PD-L2* and *FOXP3* in TCGA database. **b**, **c** The expression of PD-L2 and FOXP3 in NSCLC patient samples was detected by immunohistochemistry assay, the immunoreactive score (IRS) was calculated, and the correlation between PD-L2 and FOXP3 was analyzed. Scale bar = 50 μm. **d** The association between PD-L2 expression and clinical outcomes of the 24 sintilimab-treated NSCLC patients. **e** The association between *PD-L2* expression and clinical outcomes of 105 melanoma patients who received nivolumab immunotherapy. The data were obtained from the Gene Expression Omnibus (GEO) database (GSE91061). CR, complete response; PR, partial response; SD, stable disease; PD, progression of disease. ANOVA analysis was performed for data shown in **d** and **e**. *P < 0.05; **P < 0.01. Error bars, sem

The correlation between *PD-L2* and *FOXP3* expression levels was analyzed using The Cancer Genome Atlas (TCGA) datasets, and we found that the expression of *PD-L2* was positively correlated with *FOXP3* expression (Fig. 2a). To confirm this observation at protein level, 24 formalin-fixed paraffin-embedded tumor samples were collected from NSCLC patients treated with an anti-PD-1 antibody sintilimab (Supplementary Table 2) and examined for PD-L2 and FOXP3 expression with IHC and multiplex IHC assays. We found that the patient samples expressed variable levels of the two proteins (Fig. 2b), and a positive correlation between the two proteins was detected (Fig. 2c). Notably, PD-L2 expression level on tumors of patients achieved completed response (CR) was significantly higher than patients with stable disease (SD) and progression of disease (PD) (Fig. 2d). As to investigate the PD-L2 expression in predicting prognosis and immunotherapy benefits, we analyzed the online available resources by searching the Gene Expression Omnibus (GEO) database, and found that the data showing the PD-L2 expression level detected in 105 melanoma patients who received nivolumab immunotherapy in GSE91061 dataset was available for analysis. Consistently, the GSE91061 dataset from GEO database also indicated that melanoma patients with higher *PD-L2* expression had better clinical response to an anti-PD-1 antibody nivolumab immunotherapy (Fig. 2e). These results suggest that PD-L2 expression was associated with immunosuppressive microenvironment and clinical outcomes of the patients treated with anti-PD-1 antibodies.

### PD-L2 promotes CCL20 secretion

**Fig. 3:**
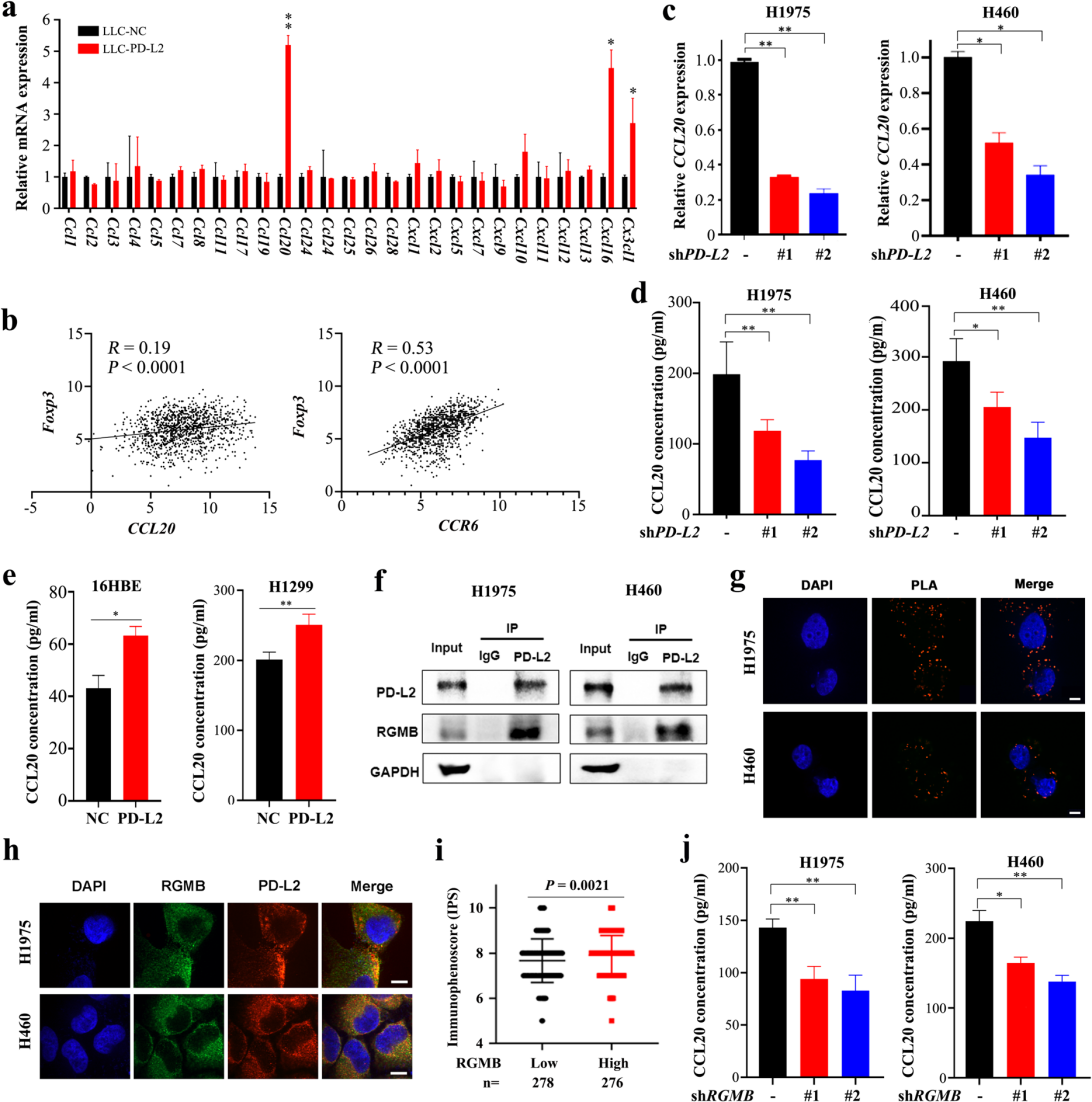
PD-L2 induces production of chemokine CCL20. **a** The mRNA expression of indicated chemokines in LLC-NC and LLC-*PD-L2* cells using qRT-PCR. Student’s *t* test. *, *P* < 0.05; **, *P* < 0.01. Error bars, sem. **b** The correlation between the expression level of *CCL20* and *FOXP3* (left), *CCR6* and *FOXP3* (right) of NSCLCs in TCGA datasets. **c** *CCL20* mRNA expression level in *PD-L2* knockdown cells was measured by qRT-PCR. **d** CCL20 concentration in supernatants of *PD-L2* knockdown H1975 (left) or H460 (right) cells, measured by ELISA assay. **e** CCL20 concentration in supernatants of *PD-L2* transfected 16HBE (left) or H1299 (right) cells, measured by ELISA assay. **f** Co-immunoprecipitation of PD-L2 and RGMB in H1975 and H460 cells transfected with exogenous *PD-L2*. **g** Proximity ligation assay (PLA) of PD-L2 and RGMB in H1975 and H460 cells. The negative control incubated with IgG antibody was shown in Supplementary Fig. 9a. **h** Confocal observation of PD-L2 and RGMB in H1975 and H460 cells after double immunofluorescent staining of PD-L2 and RGMB with indicated antibodies. The negative control incubated with IgG antibody was shown in Supplementary Fig. 9b. **i** Analysis of *RGMB* gene expression in 557 lung cancer samples downloaded from TCGA database and their immune scores using The Cancer Immunome Database (TCIA). The immune scores in *RGMB*^High^ and *RGMB*^Low^ groups were compared. The medium *RGMB* expression level was used as the cutoff value. **j** CCL20 concentration in supernatants of *RGMB* knockdown cells was measured using ELISA assay. Student’s *t* test was used unless noted otherwise, *P < 0.05; **P < 0.01. Error bars, sem

Chemokines play important roles in tumor microenvironments and can recruit immune cells to mediate immune response (Ortiz Zacarías et al. 2021). To identify chemokines that mediate PD-L2-induced Tregs enrichment in tumors, the mRNA levels of 27 chemokines were examined by qRT-PCR in LLC cells overexpressing *PD-L2*. Results showed that the expression of *Ccl20*, as well as *Cxcl16* and *Cx3cl1*, was significantly upregulated compared to that in control cells, while *Ccl20* was most significantly upregulated (Fig. 3a). In the analysis of TCGA database, we found that *CCL20* and its receptor *CCR6,* as well as *CXCL16* and *CX3CL1* were positively correlated with *FOXP3* expression in NSCLC patients (Fig. 3b, Supplementary Fig. 6). As the mRNA levels of *CCL20* was more significantly upregulated when compared to that of *CXCL16* and *CX3CL1* after overexpression of PD-L2, we further investigated the regulatory effect of PD-L2 on CCL20 expression. At cellular level, we found that stable knockdown of *PD-L2* in human NSCLC cell lines (H1975 and H460) by small hairpin RNA (shRNA) (Supplementary Fig. 7) reduced *CCL20* mRNA expression revealed by qRT-PCR assay (Fig. 3c). We tested the concentration of CCL20 in supernatants of the cells by enzyme-linked immunosorbent assay (ELISA), and found that silencing of PD-L2 reduced CCL20 concentration in H1975 and H460 cells (Fig. 3d). On the contrary, overexpression of PD-L2 in 16HBE and H1299 cells significantly increased CCL20 concentration in supernatants of the cells (Fig. 3e).

We performed co-immunoprecipitation experiments, and confirmed that PD-L2 directly interact with RGMB in H1975 and H460 cells (Fig. 3f). We conducted a Duolink proximity ligation assay (PLA) that is a technology allowing detection of protein-protein interactions in situ (at distances < 40 nm) at protein levels in the cells, and found direct binding of PD-L2 to RGMB (Fig. 3g, Supplementary Fig. 8 and 9a). Double immunofluorescent staining for PD-L2 and RGMB showed that PD-L2 and RGMB localized on the membrane with a portion localized in the cytoplasm (Fig. 3h, Supplementary Fig. 9b), possibly due to the fact that PD-L2 has a cytoplasmic domain (Latchman et al. 2001), or may suggest that the PD-L2/RGMB heterodimer could undergo internalization.

By analysis of RNA-seq data of 557 lung cancer samples downloaded from TCGA database, we divided the patients into *RGMB*^High^ and *RGMB*^Low^ groups using the median expression level as a cutoff value. After analysis of these patients’ immune scores using the Cancer Immunome Database (TCIA) (Charoentong et al. 2017), we found that *RGMB*^High^ patients had higher immunophenoscore (Fig. 3i), suggesting that these patients may be more likely to benefit from immunotherapy. We constructed a stable knockdown of RGMB in H1975 and H460 cells (Supplementary Fig. 10) and tested the effect of RGMB on CCL20 expression level, and found that stable knockdown of RGMB in these two cell lines reduced CCL20 concentration in supernatants of the cells (Fig. 3j).

**Fig. 4:**
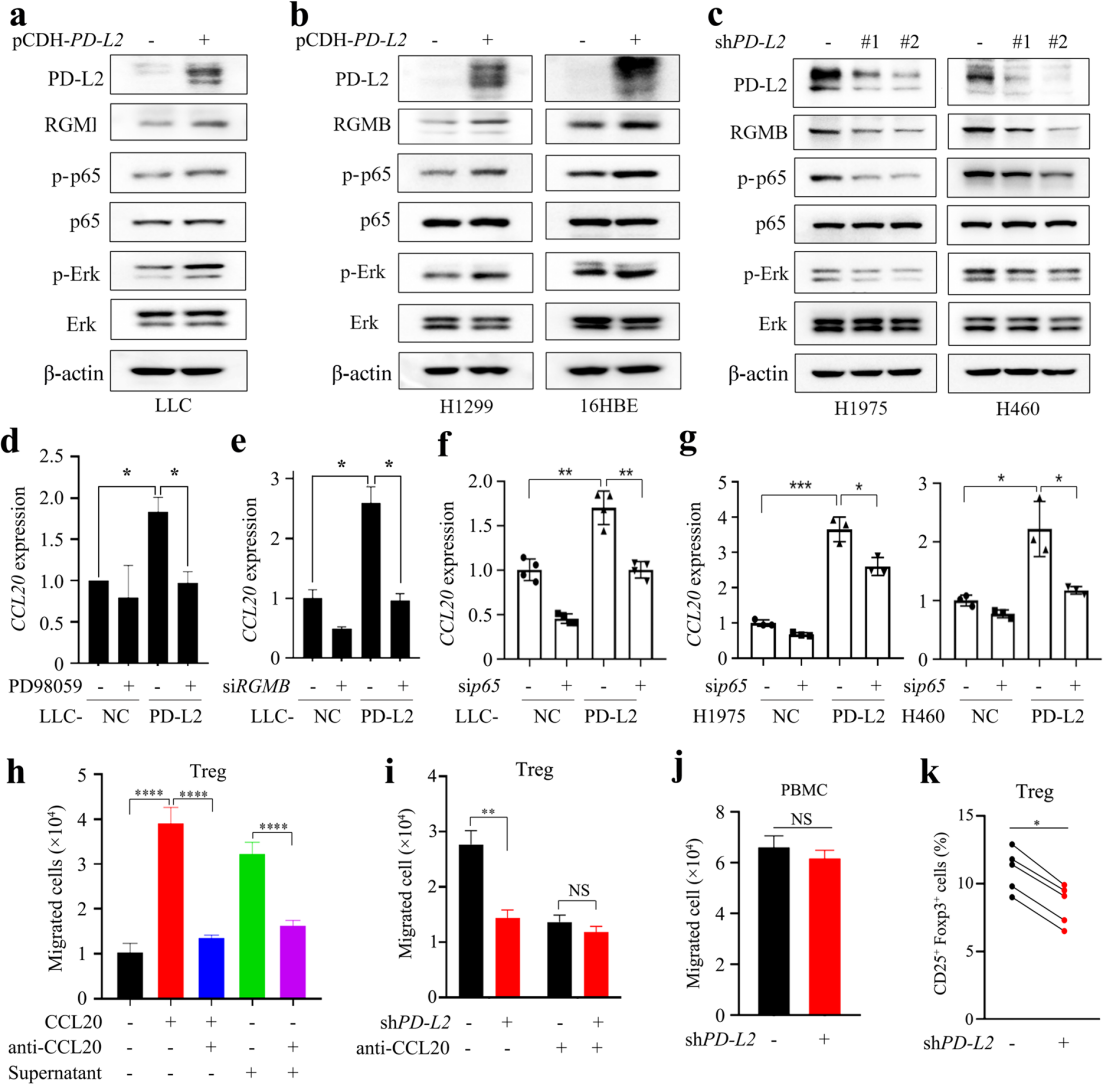
Overexpression of PD-L2 induces CCL20 secretion and Tregs recruitment. **a**, **b** Effects of ectopic expression of PD-L2 on phosphorylation of Erk and p65 in LLC, H1299 and 16HBE cells detected by western blot analysis. **c** The phosphorylation of Erk and p65 in H1975 and H460 cells after knockdown of *PD-L2*. **d** The mRNA expression of *CCL20* in LLC-NC and LLC-*PD-L2* cells in presence of an Erk inhibitor PD98059. **e** The mRNA expression of *CCL20* in LLC-NC and LLC-*PD-L2* cells after knockdown of *RGMB*. **f**, **g** The mRNA expression of *CCL20* in LLC-*PD-L2*, H1975-*PD-L2* and H460-*PD-L2* cells that were transfected with si*p65*. **h** The migration ability of Tregs after CCL20 recombinant protein or supernatants were added in the lower chamber of the transwell, in the absence or presence of CCL20 neutralizing antibody. The supernatants were taken at 24 h from pure cultures of H1299-*PD-L2* cells. **i** Effects of supernatants from *PD-L2* knockdown H1975 cells on Tregs migration ability in the absence or presence of CCL20 neutralizing antibody. **j** Effects of supernatants from *PD-L2* knockdown H1975 cells on peripheral blood mononuclear cell (PBMC) migration ability. **k** Flow cytometry analysis of the percentage of Tregs in the migrated CD4^+^ PBMC cells induced by the supernatants of *PD-L2* knockdown H1975 cells. The two points connecting by the line was referring the peripheral blood mononuclear cell (PBMC) isolated from the same volunteer. Student’s *t* test was used unless noted otherwise, *P < 0.05; **P < 0.01. Error bars, sem

The PD-L2–RGMB–BMP–BMPR oligomer is able to activate Erk (Xiao et al. 2014), which induces phosphorylation of nuclear factor κB (NFκB) p65 that controls transcription of *CCL20* (Battaglia et al. 2008; Wang et al. 2015a). To explore the molecular mechanism of PD-L2/RGMB-mediated CCL20 secretion, we analyzed the PD-L2/RGMB downstream signaling pathways and found that overexpression of PD-L2 promoted the phosphorylation of Erk (p-Erk) and p65 (p-p65) in LLC (Fig. 4a), H1299, 16HBE (Fig. 4b), H1975 and H460 cells (Supplementary Fig. 11). On the contrary, knockdown of PD-L2 in H1975 and H460 cells downregulated p-Erk and p-p65 expression levels (Fig. 4c). We found that ectopic expression of PD-L2 induced upregulation of *Ccl20* in LLC cells, while treatment of cells with an Erk inhibitor PD98059 suppressed the increase of *Ccl20* expression in LLC-PD-L2 cells (Fig. 4d). Consistently, treatment of cells with PD98059 also decreased p-p65 in PD-L2 overexpressed H1975 and H460 cells (Supplementary Fig. 11). In LLC-PD-L2 cells, silencing RGMB by si*RGMB* also inhibited *Ccl20* expression in LLC-PD-L2 cells (Fig. 4e). Knockdown of RGMB in H1975 and H460 cells downregulated p-Erk and p-p65 levels in PD-L2 overexpressed cells (Supplementary Fig. 12). Moreover, knockdown of p65 in both NC group and PD-L2-ovexpressed group (Supplementary Fig. 13a) decreased *CCL20* mRNA expression (Fig. 4f). Knockdown of p65 also resulted in downregulation of *CCL20* expression in H1975 and H460 cells (Fig. 4g, Supplementary Fig. 13b-c). Collectively, these data suggest that the PD-L2/RGMB/Erk/NFκB axis may regulate the expression of CCL20 in NSCLC, and PD-L2/RGMB direct binding on plasma membrane may be required for the phosphorylation of Erk and p65.

### PD-L2 regulates the migration of Tregs via CCL20

Chemokines regulate tumor progression by recruiting chemotactic immune cells or immunosuppressive cells to the tumor microenvironment. Specifically, CCL20 has been reported to recruit chemotactic Tregs towards tumor microenvironment to promote tumorigenesis (Wang et al. 2015a). Therefore, we explored whether PD-L2 affects chemotaxis of Tregs through CCL20. Human peripheral blood mononuclear cells (PBMCs) were isolated from heparinized peripheral blood of healthy volunteers, and the Treg cells were isolated from PBMCs by using flow cytometric cell sorting. The follow-up transwell assay demonstrated that recombinant CCL20 or supernatants taken at 24 h from pure cultures of PD-L2-overexpressed H1299 cells in the lower chamber promoted more migrated Tregs into the chamber than that of the control group, while neutralizing CCL20 with an anti-CCL20 antibody significantly reduced this migration ability (*P* < 0.001, Fig. 4h). Consistently, knockdown of PD-L2 in H1975 cells significantly reduced the migration percentage of Tregs (Fig. 4i). PBMC migration ability towards CCL20 was tested, but no significant difference was observed between migrated cells induced by shNC-H1975 cell supernatants and sh*PD-L2* H1975 cell supernatants (Fig. 4j). However, the percentage of Tregs in the migrated CD4^+^ PBMCs induced by the supernatants of PD-L2-knockdown H1975 cells was significantly decreased when compared with that induced by shNC H1975 cell supernatants (Fig. 4k). These results suggest that PD-L2 enhances migration ability of Tregs through CCL20.

### PD-L2 is highly expressed in smoker patients

To determine the PD-L2 expression level in tumor samples from NSCLCs, 45 lung cancer specimens and paired adjacent normal lung tissues were collected from treatment-naïve patients with NSCLC (Table 1). The tissues were lysed, the lysates were subjected to western blot analysis, and the relative PD-L2 expression values to β-actin determined by densitometry analysis was evaluated. We found that PD-L2 was increased (PD-L2_tumor_/PD-L2_normal_ > 1) in 68.9% (31/45) of lung cancer specimens compared to their counterpart normal lung tissues (Supplementary Fig. 14, Table 1), while the percentage of PD-L2 overexpression in smoker patients reached 14/17 (82.4%), much higher than that in non-smoker patients (6/15, 40%; Table 1). These results suggest that tobacco smoke may have a role in PD-L2 overexpression in NSCLC.

**Table 1:** Summary of baseline demographic characteristics of the 45 patients

Characteristics	Cases, n	PD-L2-high, n (%)	*P* values*
Total number	45	31 (68.9)	
Age			
<65		15 (60)	0.283
≥65	6	5 (83.3)	
not determined	14	11 (78.6)	
Gender			
male	21	15 (71.4)	0.149
female	11	5 (45.5)	
not determined	13	11 (84.6)	
Smoking			
smoker	17	14 (82.4)	0.014
non-smoker	15	6 (40)	
not determined	13	11 (84.6)	
Histology			
adenocarcinoma	18	10 (55.6)	0.543
Squamous-cell carcinoma	12	8 (66.7)	
Others or not determined	15	13 (86.7)	
TNM stage			
I-II	16	8 (50)	0.144
III-IV	16	12 75)	
not determined	13	11 (84.6)	

* Tested by the Fisher exact test

### Tobacco carcinogen induces the overexpression of PD-L2 on lung epithelial cells

**Fig. 5:**
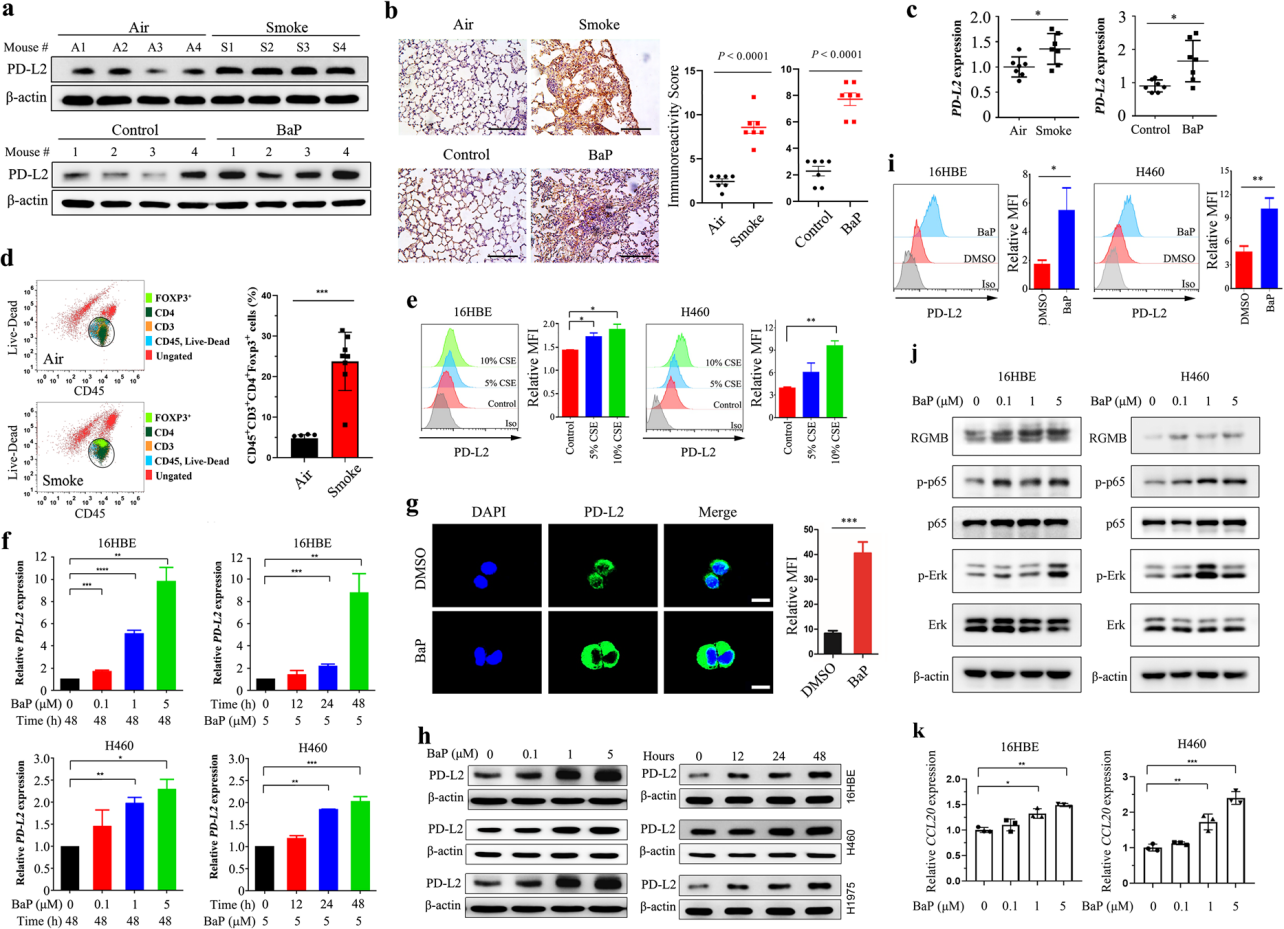
Cigarette smoke induces upregulation of PD-L2 in lung epithelial cells. **a**-**c** A/J mice were treated with cigarette smoke for 60 days and BaP for 5 weeks (*n* = 7 per group), and PD-L2 expression of lung tissue was measured by western blot, immunohistochemistry (IHC), qRT-PCR assays on the 60^th^ day. Quantitative analyses for the IHC staining of PD-L2 in lung tissues, and the scale bar in **b** is 100 μm. **d** C57BL/6 mice were exposed to cigarette smoke for 60 days and lung tissues were collected for flow cytometry analysis of FOXP3^+^ Treg cell infiltration (*n* = 8 per group). The representative scatter plots for each group were presented. **e** The cells were treated with cigarette smoke extract (CSE) at indicated concentrations for 48 h, and PD-L2 protein expression was tested by flow cytometry in 16HBE and H460 cells. **f** The cells were treated with BaP at indicated concentrations and time points, and *PD-L2* mRNA expression was assessed by qRT-PCR. **g** Immunofluorescence analysis of PD-L2 expression in 16HBE cells treated with 5 μM of BaP for 48 h. The quantitative analysis of the mean fluorescence intensity (MFI) in the cells was shown. The scale bar = 10 μm. **h** The cells were treated with BaP at indicated concentrations and time points, and PD-L2 protein expression level was tested by western blot. **i** PD-L2 expression was assessed by flow cytometry in cells treated with BaP. **j** Cells were treated with BaP at indicated concentrations, and the indicated protein expression level was tested by western blot. **k** *CCL20* mRNA expression level in cells treated with BaP was measured by qRT-PCR. ANOVA analysis was performed for data shown in **f** and **k**, and Student’s *t* test was used unless noted otherwise, *P < 0.05; **P < 0.01. Error bars, sem

We tested the effect of cigarette smoke on PD-L2 expression *in vivo*, and found that PD-L2 in lung tissues of mice exposed to cigarette smoke for 60 days was higher than that in mice exposed to clean air, as detected by western blot assays (Fig. 5a). These results were confirmed by IHC assay of lung tissue isolated from the mice exposed to cigarette smoke or clean air (Fig. 5b). Tobacco smoke also upregulated *PD-L2* at mRNA level in lung tissues of the mice (Fig. 5c). More importantly, we treated the mice with cigarette smoke or clean air exposure for 60 days, and found increased FOXP3^+^ Treg cell infiltration (Fig. 5d, Supplementary Fig. 15a-b), CCL20 mRNA expression (Supplementary Fig. 15c) and PD-L2 expression (Supplementary Fig. 16) in the lung tissues of cigarette smoke-exposed mice when compared with that in clean air-exposed mice. In the cellular level, we used cigarette smoke extract (CSE) (Wang et al. 2019, 2021) to treat 16HBE and H460 cells, which were detected for PD-L2 expression with flow cytometric immunofluorescence assay. Results showed that CSE caused upregulation of PD-L2 on the cells in a dose-dependent manner (Fig. 5e).

BaP is able to upregulate PD-L1, which has 66% of non-identity to PD-L2 (Lázár-Molnár et al. 2008), on lung epithelial cells (Wang et al. 2019). We tested the effect of BaP on PD-L2 expression, and found that this carcinogen upregulated PD-L2 in lung tissues of mice treated with BaP at both protein (Fig. 5a) and mRNA (Fig. 5c) levels. At the cellular level, BaP significantly promoted PD-L2 expression in a dose- and time-dependent manner in 16HBE and H460 cells at both mRNA level (Fig. 5f) and protein level (Fig. 5g-i). BaP and CSE also upregulated PD-L1 on lung epithelial cells (Supplementary Fig. 17a-b), while knockdown of PD-L1 did not affect BaP- or CSE-induced PD-L2 upregulation (Supplementary Fig. 17c). In addition, BaP induced upregulation of the phosphorylated Erk (p-Erk) and p-p65 in these two cells (Fig. 5j), and increased *CCL20* mRNA expression (Fig. 5k). Furthermore, we intravenously injected LLC cells into C57 mice, treated them with 100 mg/kg BaP for 3 weeks, and analyzed the FOXP3^+^ Treg cells in the lung tissues of the mice. Similarly to that shown in Fig. 5d and Supplementary Fig. 15, a higher percentage of FOXP3^+^ Treg cell was found in the BaP-treated mice than that in control mice (Supplementary Fig. 18). These results indicate that BaP is responsible for tobacco-induced upregulation of PD-L2 and increased Treg cell infiltration in NSCLC.

### BaP regulates PD-L2 expression through AhR

**Fig. 6:**
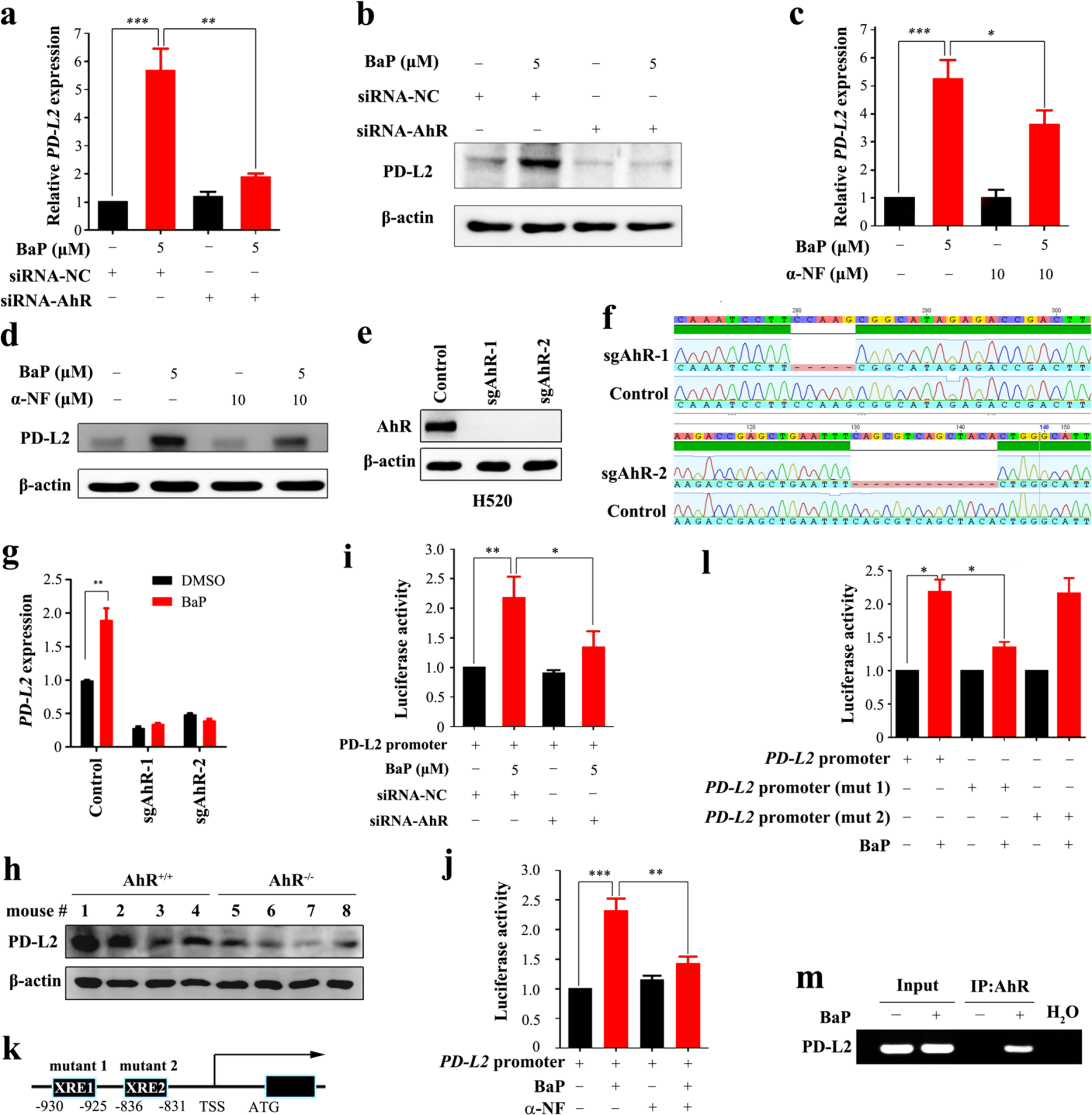
BaP induces PD-L2 expression in an AhR-dependent manner. **a**, **b** 16HBE cells were transfected with si*AhR* in the absence or presence of 5 μM of BaP, and *PD-L2* expression was measured by qRT-PCR and western blot. **c**, **d** 16HBE cells were treated with an AhR inhibitor α-NF in the absence or presence of 5 μM of BaP, and PD-L2 expression was tested by qRT-PCR and western blot. **e**, **f** Construction of AhR knockout H520 cell line, and knockout efficiency was examined by western blot and validated by Sanger sequencing. **g** *PD-L2* mRNA expression in the AhR knock-out cell line was measured by qRT-PCR after treatment with BaP. **h** Western blot analysis of PD-L2 expression in lung tissues of *AhR*^*+/+*^ and *AhR*^*-/-*^ mice. **i**, **j** H460 cells transfected with the *PD-L2* promoter were treated with BaP for 48 h after *AhR* knockdown or in the presence of α-NF, and luciferase activity was measured. **k**, **l** H460 cells transfected with the wild-type or mutant *PD-L2* promoters were treated with BaP for 48 h, and luciferase activity was measured. **m** ChIP assay was performed using AhR-precipitated DNA samples from the cells treated with or without BaP. The expression of *PD-L2* was evaluated by qRT-PCR, and the primers were used to detect XRE1. Student’s *t* test was used unless noted otherwise, *P < 0.05; **P < 0.01. Error bars, sem

Aryl hydrocarbon receptor (AhR) is a receptor of BaP that can bind to the xenobiotic-responsive element (XRE) or aryl hydrocarbon responsive element (AHRE) to regulate its target genes (Chen et al. 2021), and mediates BaP-induced upregulation of PD-L1 on lung epithelial cells (Wang et al. 2019). We investigated the role of AhR in tobacco-induced PD-L2 overexpression. By knockdown of AhR (Supplementary Fig. 19), we found that PD-L2 expression induced by BaP was significantly reduced as determined by qRT-PCR (Fig. 6a) and western blot (Fig. 6b). In addition, the upregulation of PD-L2 induced by BaP was inhibited by a synthetic AhR inhibitor α-naphthoflavone (α-NF) at both mRNA (Fig. 6c) and protein levels (Fig. 6d). To further explore the molecular mechanism of AhR in regulation of PD-L2, we constructed AhR knockout cell line by using CRISPR/Cas9 technique, and tested the gene knockout efficiency by western blot (Fig. 6e) and Sanger sequencing (Fig. 6f) analyses. We found that BaP significantly induced PD-L2 mRNA and protein expression as well as *CCL20* mRNA expression in control cells (*P* < 0.01), but not in AhR knockout cells (Fig. 6g, Supplementary Fig. 20-21). Consistently, the expression of PD-L2 in AhR deficient mice was significantly lower than that in wild type mice after treatment with BaP (Fig. 6h). The luciferase activity assay showed that BaP promoted the transcriptional activity of the *PD-L2* promoter, while silencing of AhR (Fig. 6i) or adding the AhR inhibitor α-NF (Fig. 6j) significantly suppressed this activity. By analyzing the sequence of *PD-L2* by using the JASPAR software, we found two XRE-like sites for AhR binding in the *PD-L2* promoter region (Fig. 6k). We found that BaP increased wild-type *PD-L2* promoter-driven luciferase activity, while deletion of the XRE1 significantly inhibited the luciferase activity (*P* < 0.05, Fig. 6l). Further chromatin immunoprecipitation (ChIP) was performed to test AhR-*PD-L2* interaction. We found that AhR directly bound to the *PD-L2* promoter (Fig. 6m). The IHC staining of FOXP3^+^ cells showed a higher staining intensity in the lung tissues of the AhR^+/+^ mice than that in AhR^-/-^ mice (Supplementary Fig. 22). Collectively, these results suggest that AhR mediates BaP-induced PD-L2 expression in lung cancer cells.

## Discussion

Immune checkpoint inhibitors (ICIs) targeting PD-1/PD-L1 axis have been widely used in treating human solid and liquid tumors (Morad et al. 2021). However, responses to anti-PD-1/anti-PD-L1 treatment vary from patients who have long-term remission, to those who have rapid disease progression (Weiss et al. 2021). Accumulated evidence has shown that the tumor cells have a low PD-L2 expression when compared to PD-L1, however PD-L2 binding affinity to PD-1 is higher than that of PD-L1 (Ghiotto et al. 2010). Therefore, the effects of PD-L2 on tumor progression cannot be neglected. A previous study found that PD-L2 expression in tumor cells significantly suppressed anti-tumor immune responses by exhausting CD8^+^ T cells in renal cell carcinoma (RCC) and lung squamous cell carcinoma (LUSC), which contributes to development of resistance to anti-PD-L1 treatment. However, this resistance was overcome by an anti-PD-1 monoclonal antibody or combined treatment with an anti-PD-L2 antibody (Tanegashima et al. 2019). This immune checkpoint blockade resistance could also be reversed by blocking the PD-1/PD-L2 pathway in ovarian cancer (Miao et al. 2021). In addition, clinical response to pembrolizumab in patients with head and neck squamous cell carcinomas was partly related to blockade of PD-1/PD-L2 axis, and PD-L2 positivity was significantly associated with overall response rate (ORR) regardless of PD-L1 status (Yearley et al. 2017). Recently, PD-L2 has been suggested as a potential therapeutic target in prostate cancer after characterizing the immune landscape of prostate cancer (Zhao et al. 2019). Meanwhile, immunohistochemical evaluation of PD-L2 expression in NSCLC specimens showed that PD-L2 expression may be a potential biomarker for response to PD-1/PD-L1-targeted immunotherapy (Matsubara et al. 2019). In this study, we found that lung cancer patients with high PD-L2 expression showed better treatment response to an anti-PD-1 antibody sintilimab than those patients with low PD-L2 expression. Bagaev et al. developed a multi-omics and robust analytical platform to classify the entire tumor composition, and defined four distinct tumor microenvironment subtypes predictive of response to immunotherapy based on melanoma that were conserved across at least 20 additional cancers, including lung cancer (Bagaev et al. 2021), suggesting the likely similar tumor microenvironment in melanoma and lung cancer. The GEO dataset (GSE91061) we used in this study is commonly utilized to investigate certain prognostic signatures of genes in predicting patient response to the immune checkpoint blockade therapy. Similarly, analysis of this database also indicated that melanoma patients with higher PD-L2 expression had better clinical response to nivolumab immunotherapy. These findings suggest that PD-L2 may play a role in clinical responses observed with anti-PD-1 therapy.

Previous studies have reported the clinical significance of PD-L2 expression in a variety of tumor types, including lung cancer (Solinas et al. 2020). However, the results obtained from different research groups were not consistent. Shinchi et al. observed longer progression-free survival (PFS) in lung adenocarcinoma patients with positive PD-L2 expression (Shinchi et al. 2019), while another retrospective study reported that the high expression of PD-L2 on tumor cells was associated with a poor prognosis for patients (Takamori et al. 2019). Meanwhile, several studies found that PD-L2 expression was an independent prognostic factor associated with a good prognosis in oropharyngeal squamous cell carcinoma (Danilova et al. 2016; Obeid et al. 2016; Steuer et al. 2018) and melanoma (Danilova et al. 2016; Obeid et al. 2016; Steuer et al. 2018) patients. However, colorectal cancer, malignant salivary gland tumor, and chromophobe cell carcinoma patients with high expression of PD-L2 showed poor prognostic outcomes (Danilova et al. 2016; Obeid et al. 2016; Steuer et al. 2018). This discrepancy might be attributed to differences in sample sizes and study settings. Our findings suggested that higher PD-L2 expression was observed in tumor tissues than that in normal tissues, and was associated with worse outcomes in NSCLC patients, especially in smokers. The molecular mechanism study revealed that cigarette smoke and carcinogen BaP could increase AhR-mediated PD-L2 expression. Moreover, a recent meta-analysis also showed that high PD-L2 expression indicated poor overall survival and disease-free survival in lung cancer patients, and was associated with smoking status (Lin et al. 2021), which was consistent with our current findings.

In addition to its inhibitory function on T cells through PD-1/PD-L2 interaction, PD-L2 also exerts immune regulatory effect via binding to its costimulatory receptor RGMB. This second binding partner of PD-L2 was discovered to have a binding affinity similar to PD-L2-PD-1 (Pauken et al. 2021; Xiao et al. 2014). It has been reported that activation of PD-L2/RGMB pathway induces osteosarcoma growth and lung metastasis (Ren et al. 2019), and PD-L2 stimulates CD4^+^ T-helper 1 response through PD-L2/RGMB interaction (Nie et al. 2018). The binding of PD-L2 with RGMB involves binding of RGMB to BMP2/4, type I and II BMP receptors, and neogenin to form a supercomplex, which exerts various biological functions through mediating downstream signaling pathways by phosphorylation of type I BMP receptors (Xiao et al. 2014). However, it is worth noting that the role of RGMB in tumor progression remains controversial because both inhibitory and promoting functions have been reported in various experimental systems. For instance, Li et al. reported that knockdown of RGMB resulted in enhanced cell proliferation, adhesion, and migration in breast cancer cells (Li et al. 2012), while overexpression of RGMB suppressed NSCLC progression (Li et al. 2016). In this study, we found that activation of PD-L2/RGMB pathway increased CCL20 expression to promote lung cancer progression. The enhanced CCL20 secretion facilitated chemotactic migration of Tregs *in vitro* and *in vivo*. The mechanism study revealed that BaP-induced upregulation of PD-L2 promoted the phosphorylation of p65 to activate NF-κB pathway. Another tobacco carcinogen NNK (4-(methylnitrosamino)-1-(3-pyridyl)-1-butanone) also induces CCL20 production via activation of NF-κB pathway (Wang et al. 2015a), highlighting the complicated actions and mechanisms underlying tobacco smoke in inducing lung cancer. Hurrell et al. demonstrated that PD-1/PD-L2 axis induced Tregs in the lungs by increasing peripheral Treg number, FOXP3 expression, tricarboxylic acid (TCA) cycle, and mitochondrial function, and thus contributed to the development of respiratory tolerance and airway hyperactivity (Li et al. 2016). It should be noted that PD-L2 also altered CXCL16 and CX3CL1 expression at the mRNA level in this study. Further studies may be conducted to determine whether CXCL16 and CX3CL1 are also the major targets of the PD-L2/RGMB pathway. In addition, as PD-L2, RGMB, and BMP receptors form a protein complex to trigger the activation of downstream signaling pathways, the BMP receptor effects on PD-L2-induced ERK and P65 phosphorylation, as well as CCL20 expression required further investigations.

AhR, a ligand-dependent transcription factor that belongs to the basic helix-loop-helix (bHLH)/per-Arnt-sim (PAS) superfamily, plays an important role in regulating tumorigenesis including environmental lung carcinogenesis (Akhmetova et al. 2022). AhR mediates tobacco-induced PD-L1 expression on lung epithelial cells and tumor-repopulating cell-driven PD-1 upregulation in CD8^+^ T cells (Wang et al. 2019). AhR mediates BaP-induced production of a chemokine CXCL13, the knockout of which significantly inhibits BaP-initiated lung cancer (Wang et al. 2015b). Here we showed that AhR also mediates tobacco smoke/BaP-induced upregulation of PD-L2 on lung epithelial cells, which binds RGMB and activates NFκB p65 to generate CCL20, leading to recruitment of Tregs and disease progression. These results indicate that AhR has a central role in lung carcinogenesis and may serve as a therapeutic/chemopreventive target for lung cancer, confirmed by the findings that deficiency in AhR inhibited BaP-induced lung cancer *in vivo* (Wang et al. 2019). Furthermore, AhR is associated with response to immunotherapy and significantly enhanced the predictive power of PD-L1 in predicting the response of NSCLC to PD-1 blockade (Wang et al. 2019). Since PD-L2 is also a target of AhR, its role in predicting patient response to ICIs warrants to be tested, especially in combination with AhR and PD-L1.

## Supplementary information

ESM 1 Supplementary data of this work contain 22 supplementary figures and 2 supplementary table. (DOCX 9458 kb)

## Data Availability

No datasets were generated or analysed during the current study.
