# Supplementary material for: PD-L2 mediates tobacco smoking-induced recruitment of regulatory T cells via the RGMB/NFκB/CCL20 cascade
Source: Cell Biol Toxicol. 2024 Jul 23;40(1):56. doi: 10.1007/s10565-024-09892-3 (PMC11266262; doi:10.1007/s10565-024-09892-3)
Supplement: Supplementary file 1 — Supplementary data of this work contain 22 supplementary figures and 2 supplementary table. (DOCX 9458 kb) [file 10565_2024_9892_MOESM1_ESM.docx]

**Supporting information**

**PD-L2 mediates tobacco smoking-induced recruitment of regulatory T cells via the RGMB/NFκB/CCL20 cascade**

Hua Guo^1#^, Chen Zhang^1,2#^, Yu-Ke Shen^1#^, Jian-Dong Zhang^3,4^, Fu-Ying Yang^1^, Fan Liang^1,5^, Wei Wang^3^, Yu-Tao Liu^1^, Gui-Zhen Wang^1^, Guang-Biao Zhou^1*^

^1^ State Key Laboratory of Molecular Oncology & Department of Medical Oncology, National Cancer Center/National Clinical Research Center for Cancer/Cancer Hospital, Chinese Academy of Medical Sciences and Peking Union Medical College, Beijing, 100021, China;

^2^ School of Life Sciences and Engineering, Handan University, Handan, Hebei Province, 056005, China;

^3^ Department of Urology, Beijing Chaoyang Hospital, Capital Medical University, Beijing, 100020, China;

^4^ Shanxi Bethune Hospital Affiliated with Shanxi Academy of Medical Sciences, Taiyuan, Shanxi Province, 030032, China.

^5^ School of Basic Medicine, Weifang Medical University, Shandong, 261000, China.

# These authors equally contribute to this work

* Correspondence to: Guang-Biao Zhou ([gbzhou@cicams.ac.cn](mailto:gbzhou@cicams.ac.cn)).

**
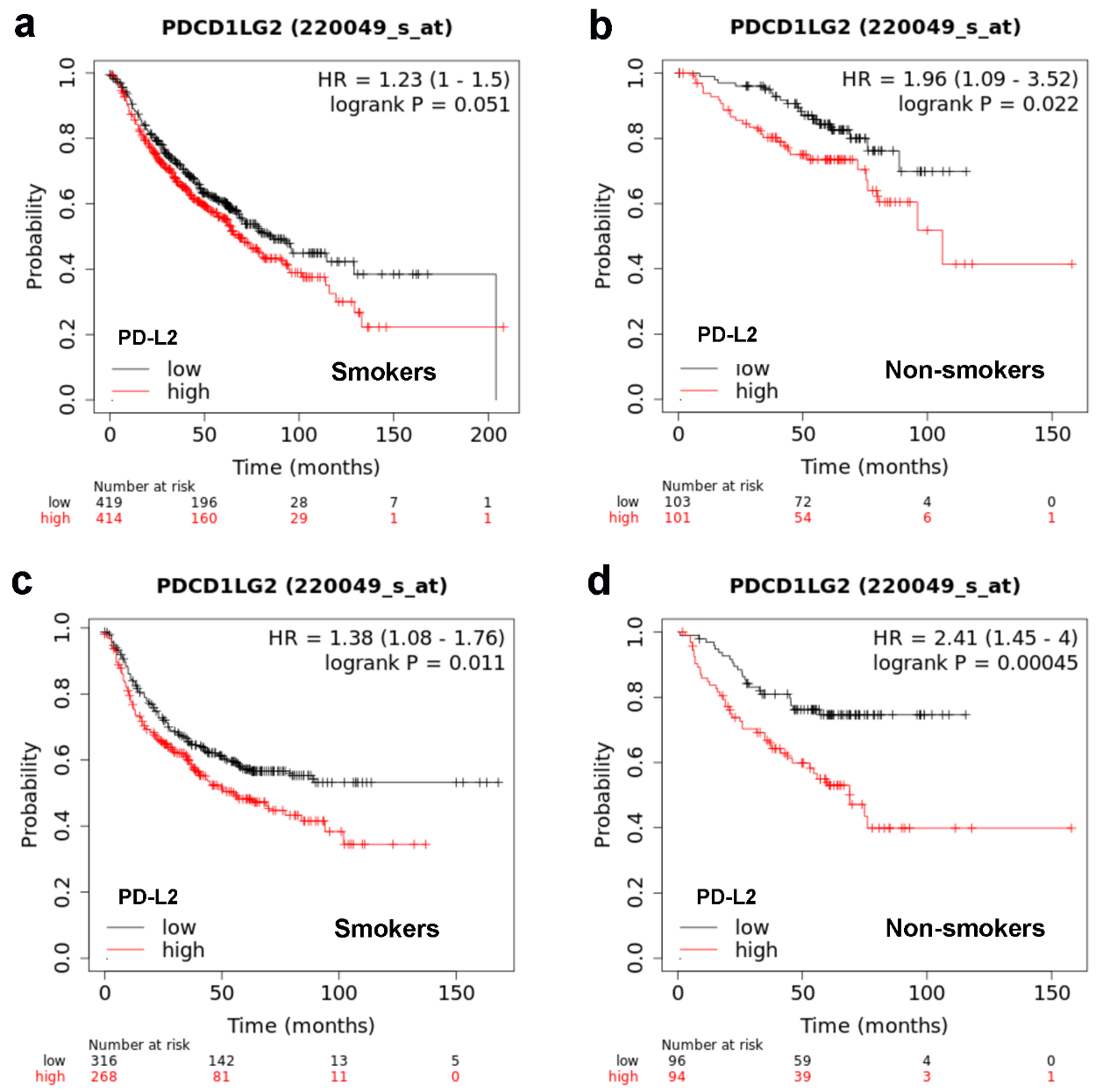
**

**Supplementary Figure 1. Analysis of the effects of PD-L2 prognostic value in NSCLC patients by using Kaplan-Meier Plotter database.** The effect of expression level of PD-L2 on overall survival curves for (**a**) smokers and (**b**) non-smokers, and time to first progression curves (FP) for (**c**) smokers and (**d**) non-smokers among the NSCLC patients were analyzed using the Online Survival Analysis Software, respectively.

**
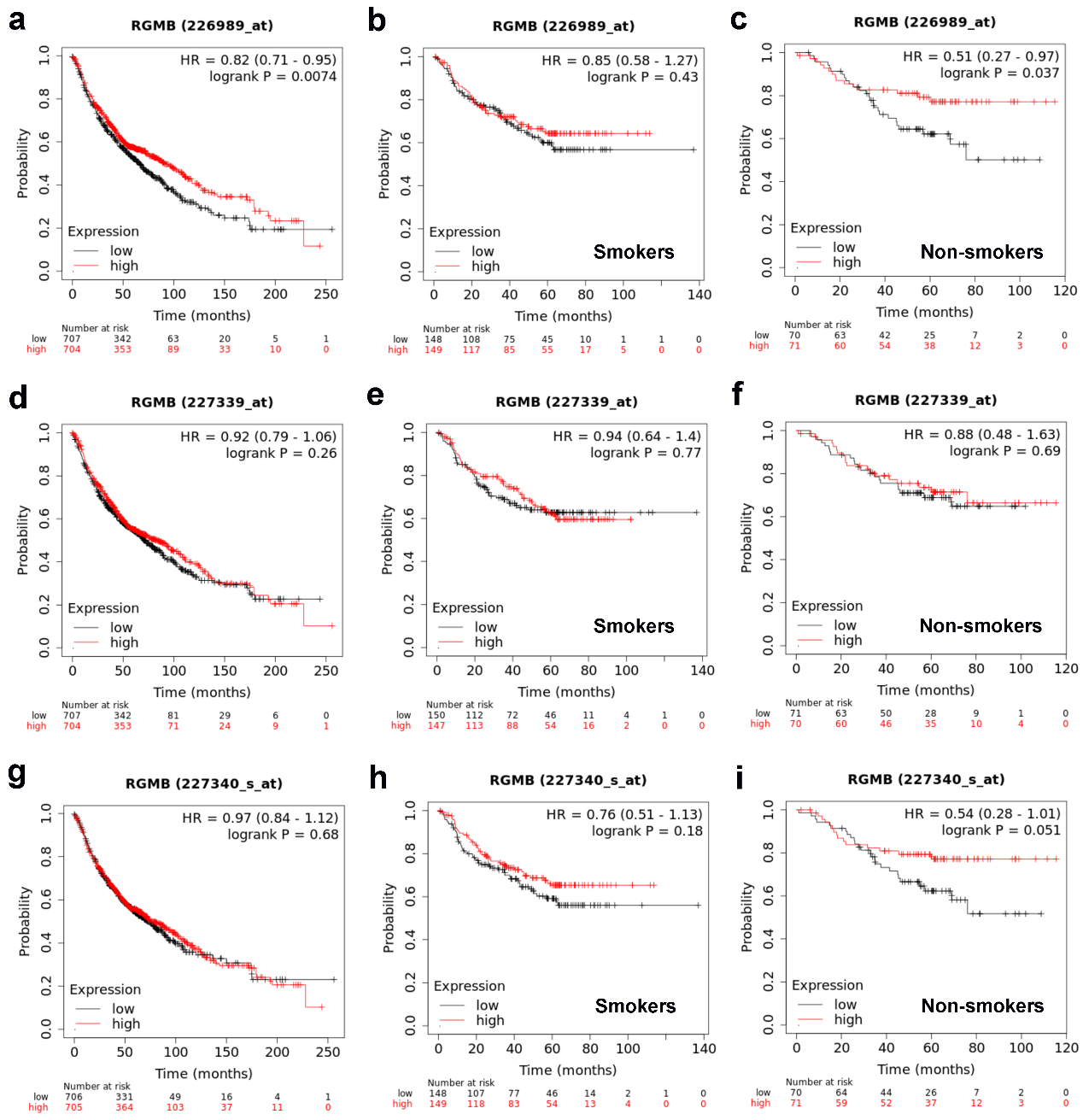
**

**Supplementary Figure 2. The expression level of RGMB was not associated with clinical outcome of NSCLC patients.** Data were obtained from the Online Survival Analysis Software (<https://kmplot.com/analysis/index.php?p=service&cancer=lung>). In this database, three RGMB probes for detection of RGMB mRNA expression were used. The results obtained from each probe for the overall survival curves and time to first progression curves (FP) for smokers and non-smokers among the NSCLC patients were shown.


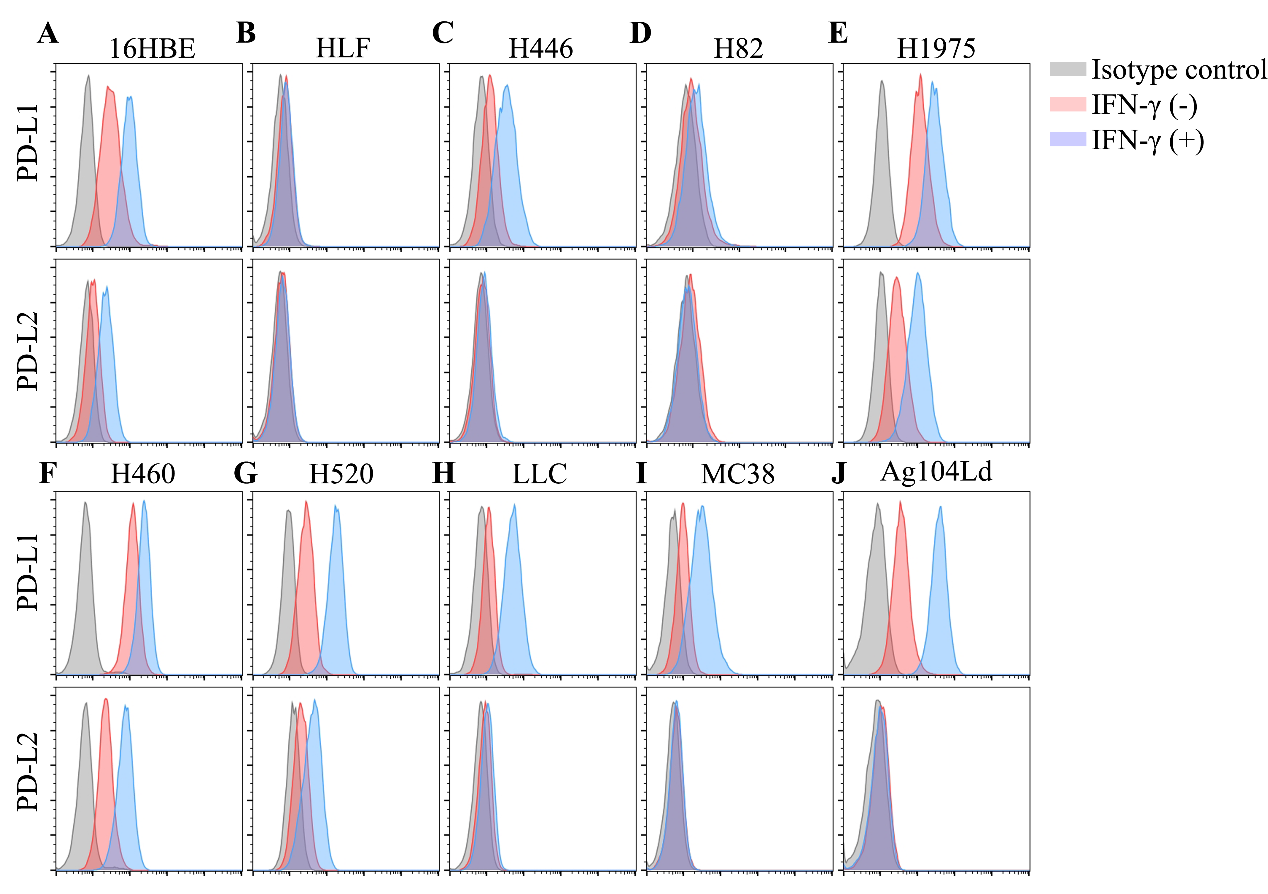


**Supplementary Figure 3. The expression of PD-L1 and PD-L2** **with or without IFN-γ treatment in indicated cell lines was examined by flow cytometry.**


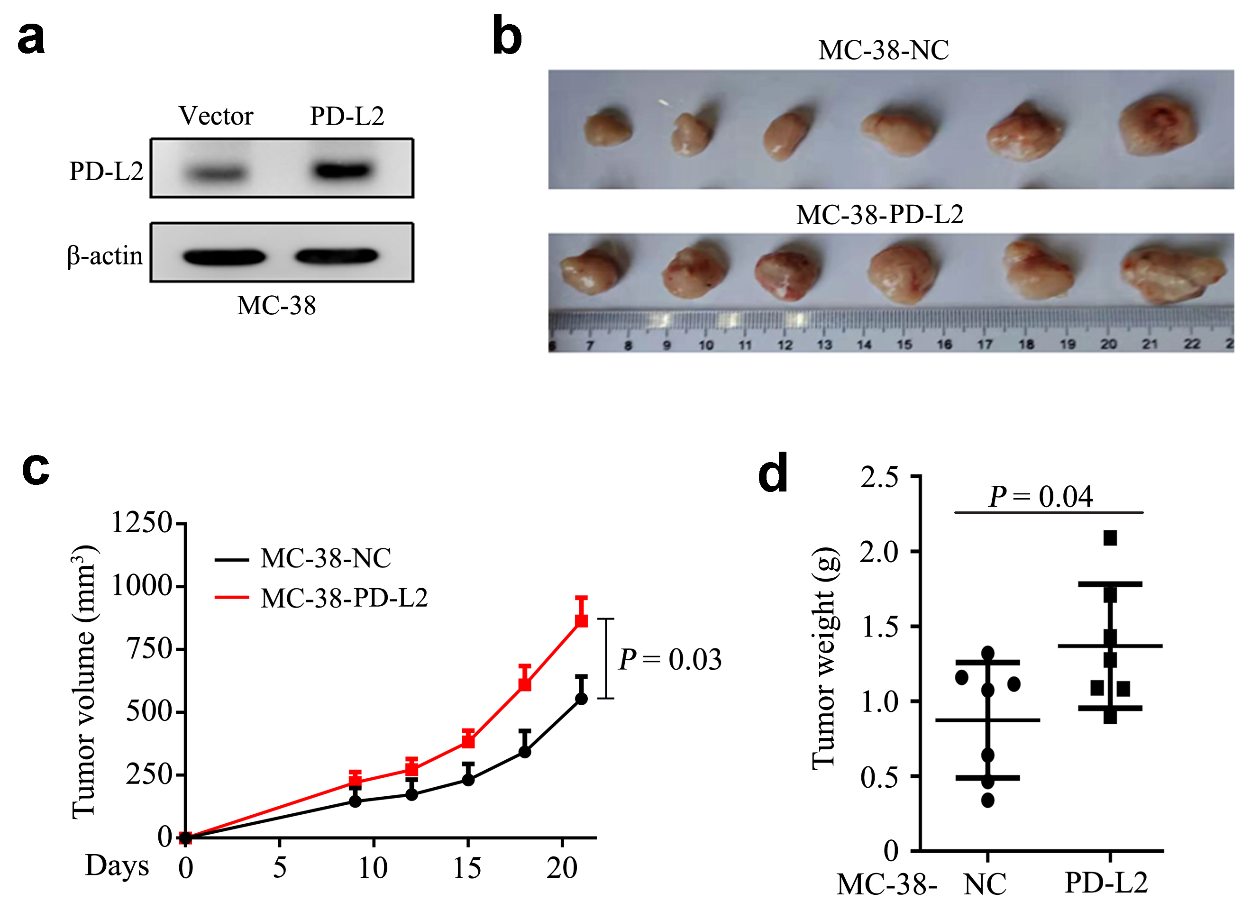


**Supplementary Figure 4. PD-L2 promotes MC38 tumor growth *in vivo*. a** Construction of PD-L2 stably overexpressed MC-38 cell line. The PD-L2 expression was examined by western blot. **b-d** Effects of PD-L2 on MC-38 xenograft murine model. The size (**b**) and weight (**d**) of tumor tissues isolated from subcutaneous tumor-bearing mice on day 30 after MC-38-NC or MC-38-PD-L2 cell inoculation. **c** Tumor volume between the MC-8-NC and MC-38-PD-L2 groups was compared on Day 22 after tumor cell innovation. Student’s *t* test. Error bars, sem.

**
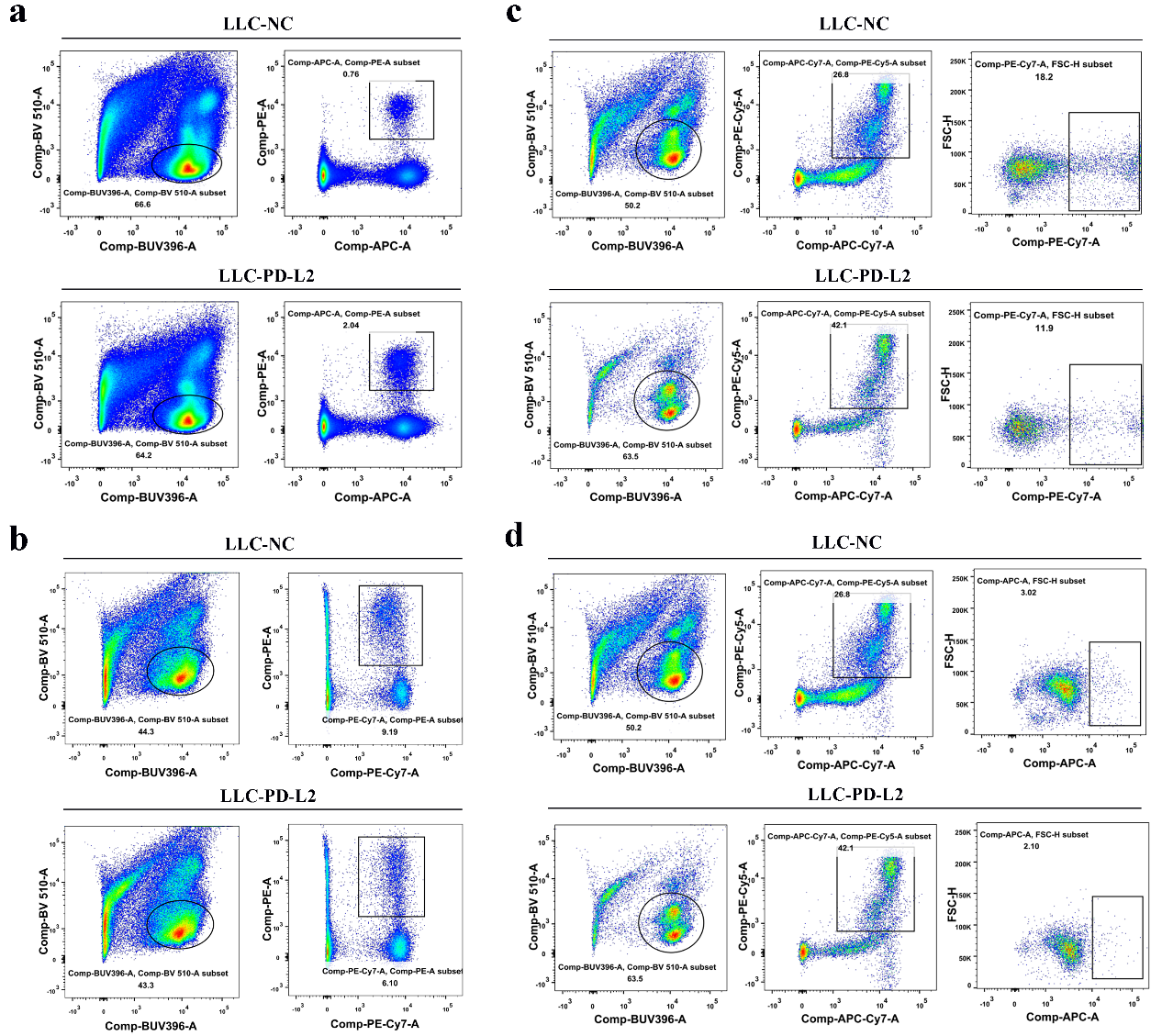
**

**Supplementary Figure 5. Representative scatter plots of** (**a**) Treg, (b) MDSC, (c) M1 and (d) TAM cells **in lung tissues of mice after LLC-NC or LLC-PD-L2 cell intravenous injection by flow cytometry analysis.** The Zombie Aqua™ dye (BV510 channel) was used to determine live cells. The Foxp3^+^ cells gating on live CD45^+^CD3^+^CD4^+^ T cells represented Treg cell, the MHCⅡ^+^ cells gating on live CD45^+^Gr1^+^ cells represented MDSC cell, the CD86^+^ cells gating on CD45^+^CD11b^+^F4/80^+^ cells represented M1 cell, and the CD206^+^ cells gating on live CD45^+^CD11b^+^F4/80^+^ cells represented TAM cell.

**
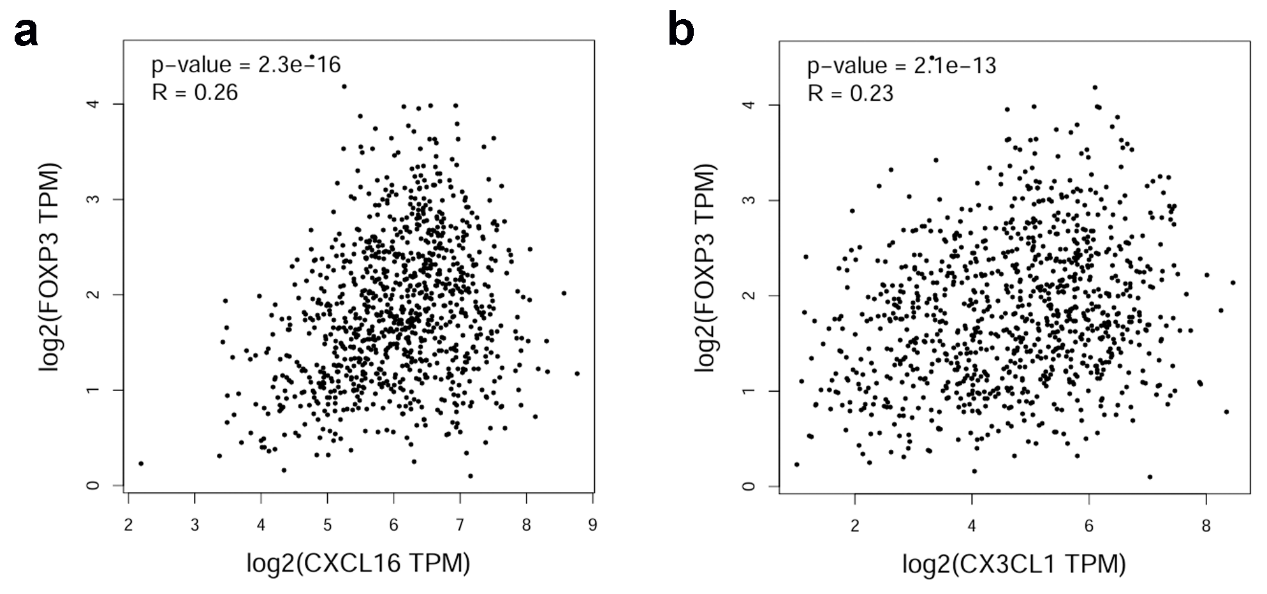
**

**Supplementary Figure 6. A correlation analysis for (a) CXCL16 and (b) CX3C11 with FOXP3 in the TCGA cohort.**

**
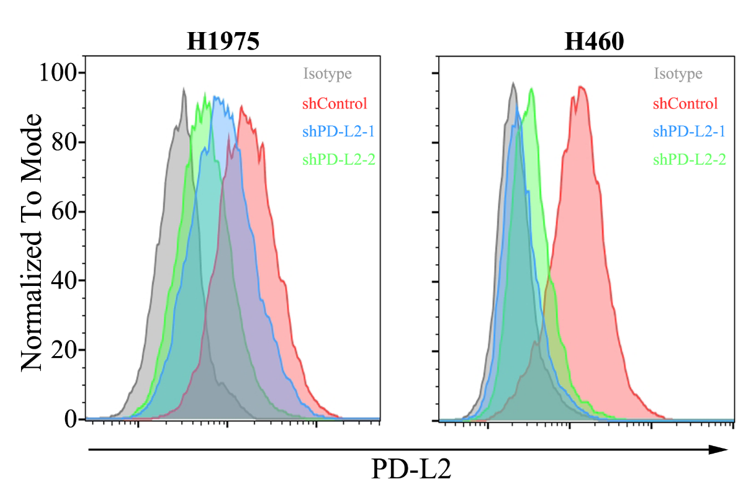
**

**Supplementary Figure 7. The PD-L2 expression in PD-L2 knockdown cells was measured by flow cytometry.**


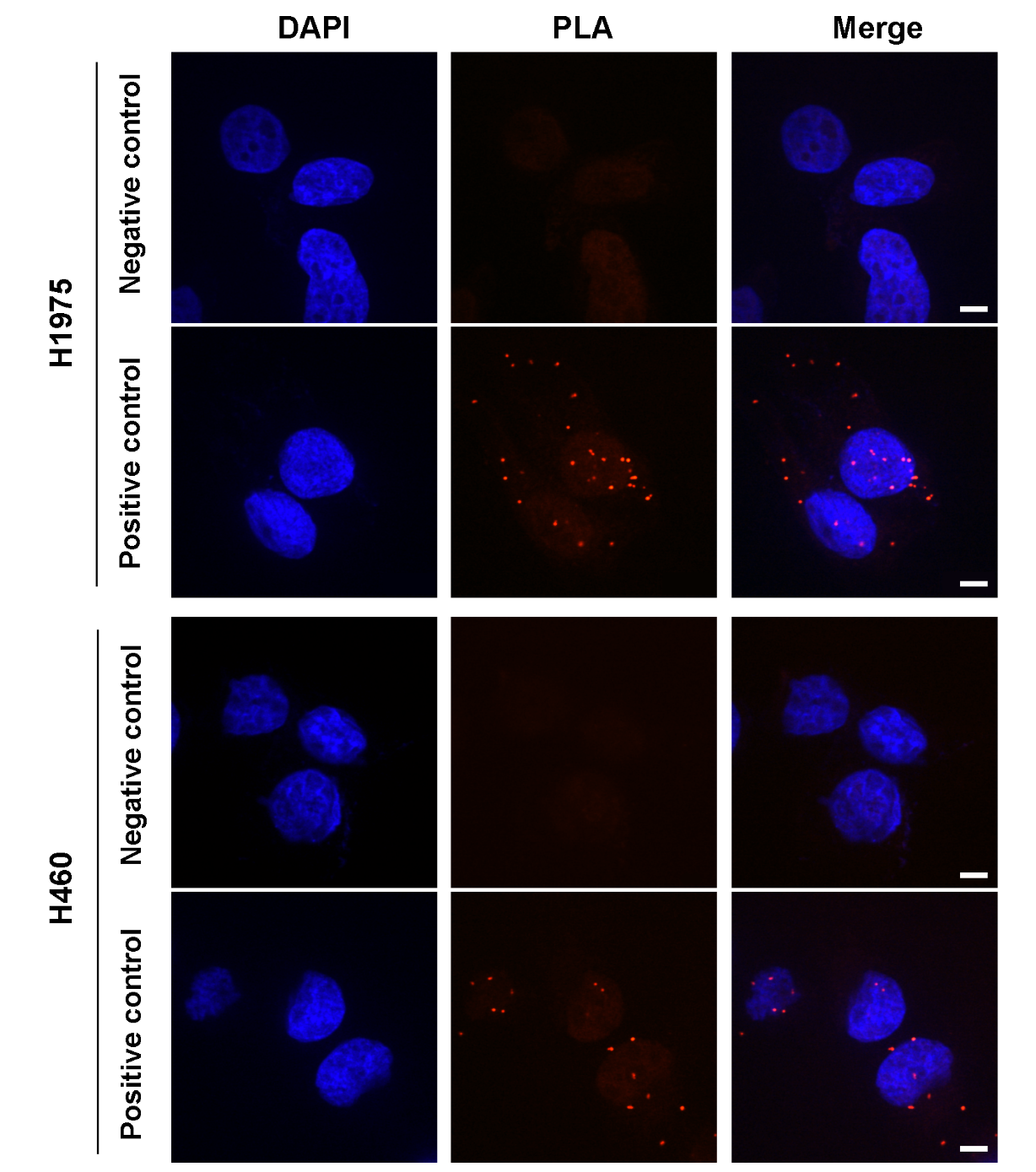


**Supplementary Figure 8. Proximity ligation assay (PLA) of detecting endogenous PD-L2 and RGMB in non-transfected H1975 and H460 cells.** The cells incubated with IgG antibody were set as negative control when compared with the cells incubated with anti-PD-L2 and anti-RGMB antibodies. Scale bars = 10 μm.

**
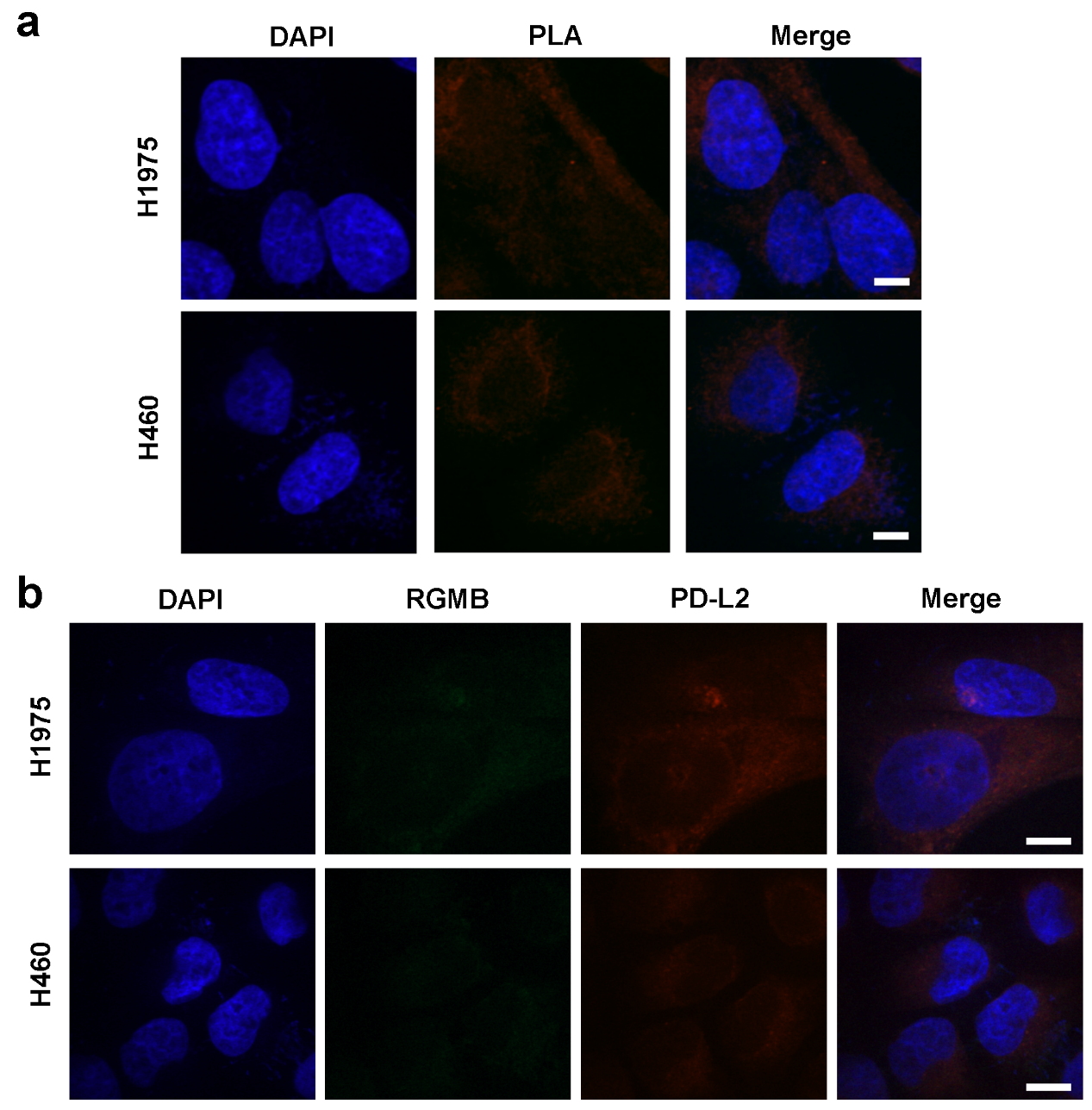
**

**Supplementary Figure 9. Negative control for (A) proximity ligation assay (PLA) and (B) double immunofluorescent staining of PD-L2 and RGMB in H1975 and H460 cells.** The cells incubated with IgG antibody were set as negative control when compared with the cells incubated with anti-PD-L2 and anti-RGMB antibodies that shown in Figure 3g and 3h, respectively. Scale bars = 10 μm.


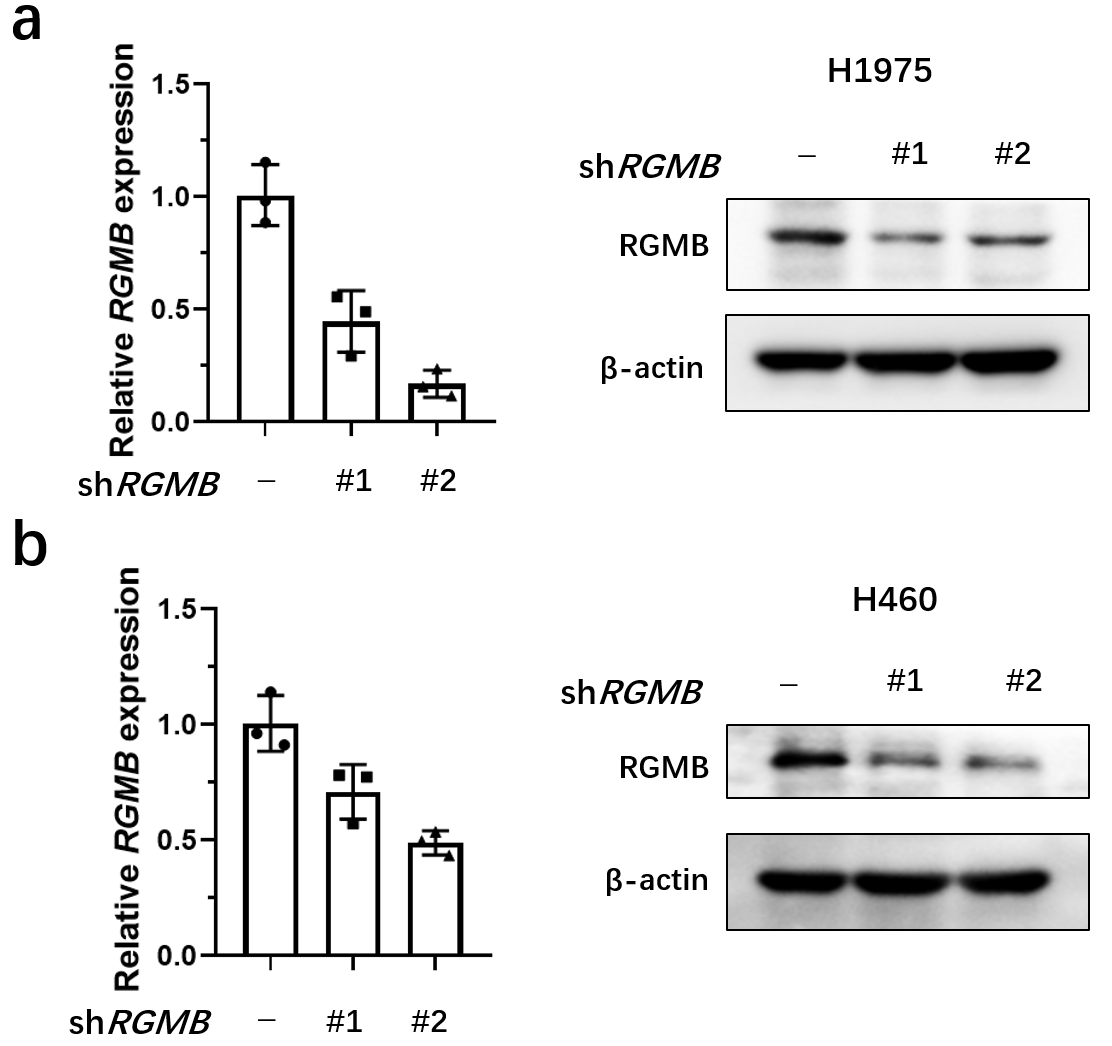


**Supplementary Figure 10. The RGMB expression in scramble- and shRGMB-expressing cells was measured by qRT-PCR and Western blot assays in (a) H1975 and (b) H460 cells.**


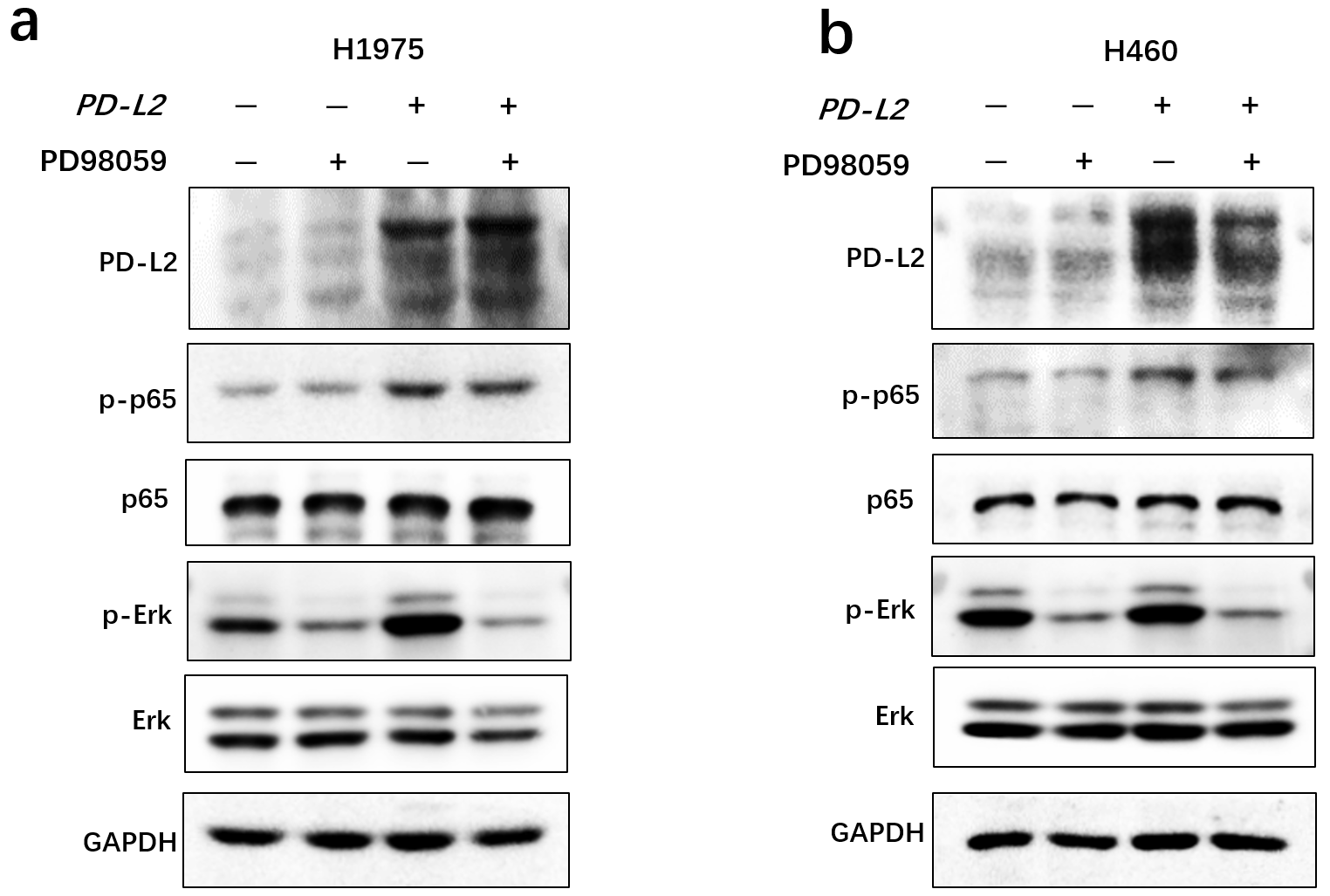


**Supplementary Figure 11. Western blot analysis of** **p65 phosphorylation in the absence or presence of the ERK inhibitor PD98059 in PD-L2-overexpressed cell lines.** H1975 and H460 cells were transfected with PD-L2 plasmid for 24 h. Then medium was refreshed, and cells were treated with 10 μM PD98059 for 24 h. After that, cells were collected for Western blot analysis.


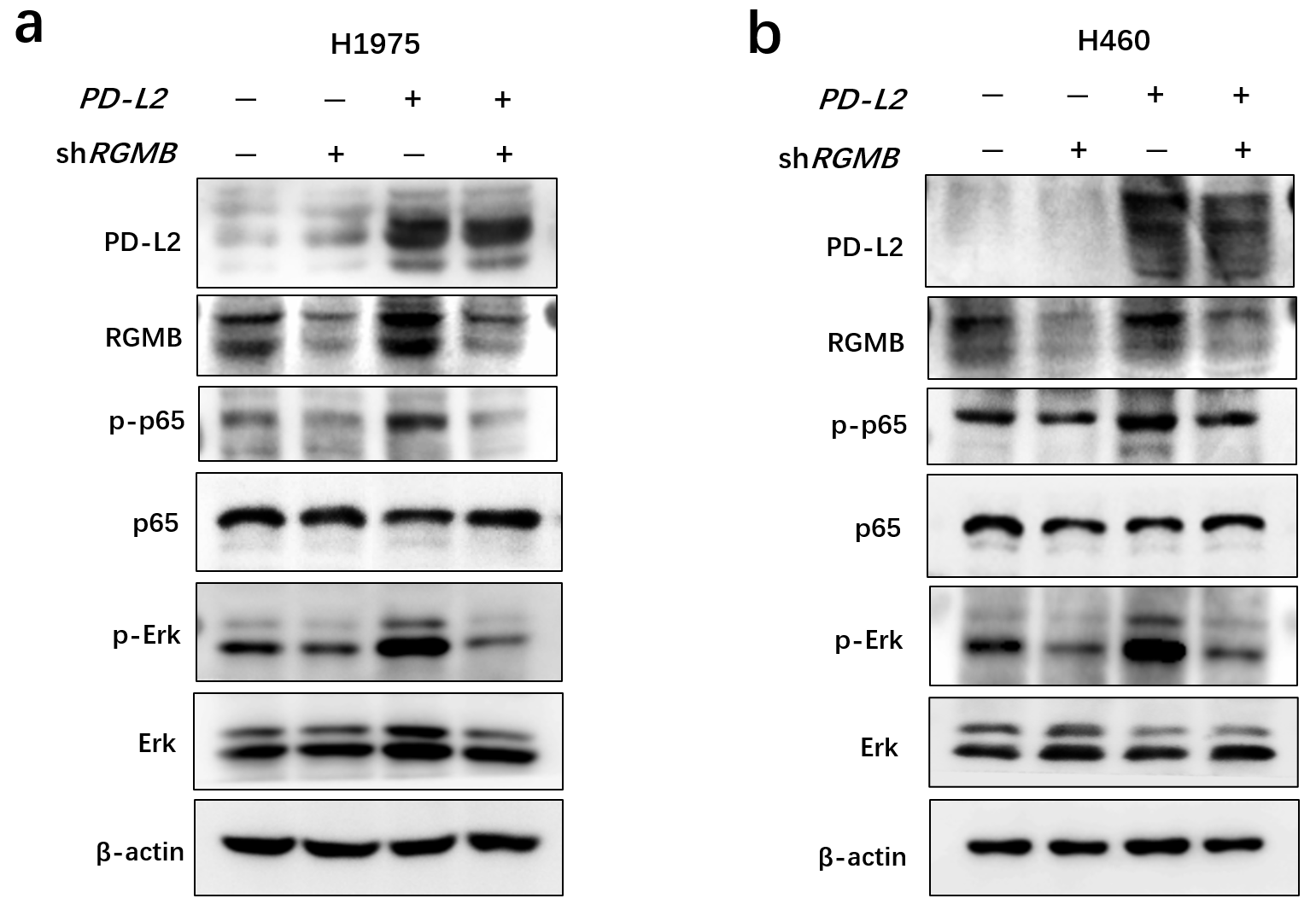


**Supplementary Figure 12. Phosphorylation of p65 and Erk in the shNC or sh*RGMB* cells overexpressing PD-L2**. The shNC or sh*RGMB*-expressing H1975 and H460 cells were transfected with PD-L2 plasmid for 48 h, and lysed subjected to Western blot analysis.


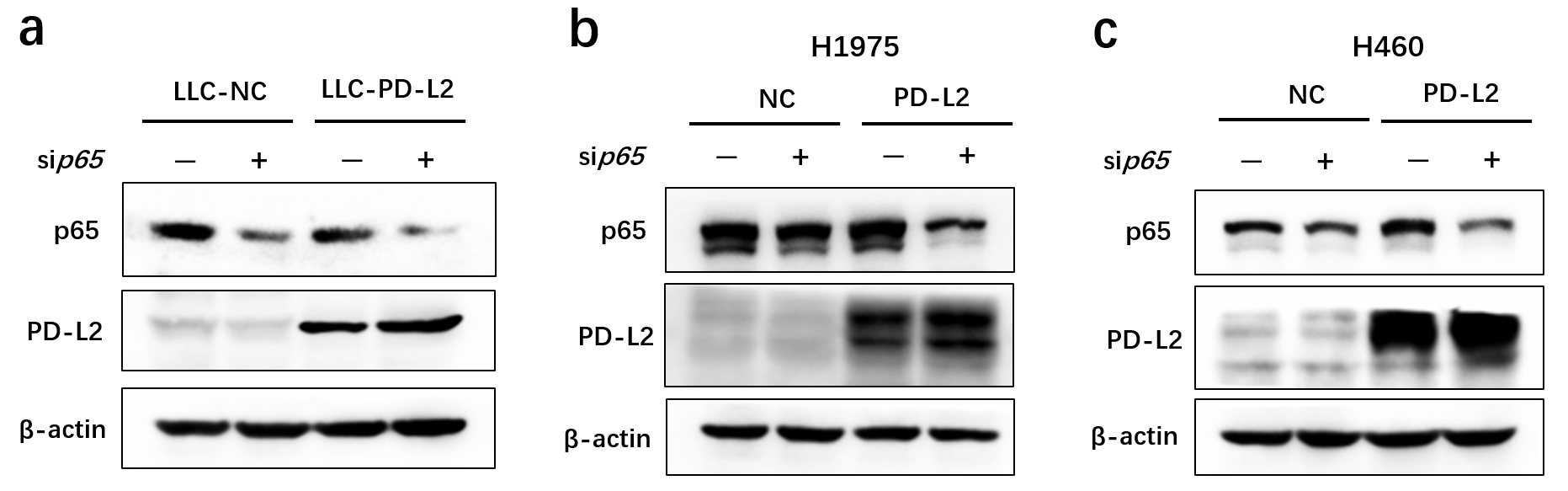


**Supplementary Figure 13. The expression of p65 and PD-L2 in LLC-PD-L2 (a), H1975-PD-L2 (b) and H460-PD-L2 (c) cells transfected with or without si*p65*.**


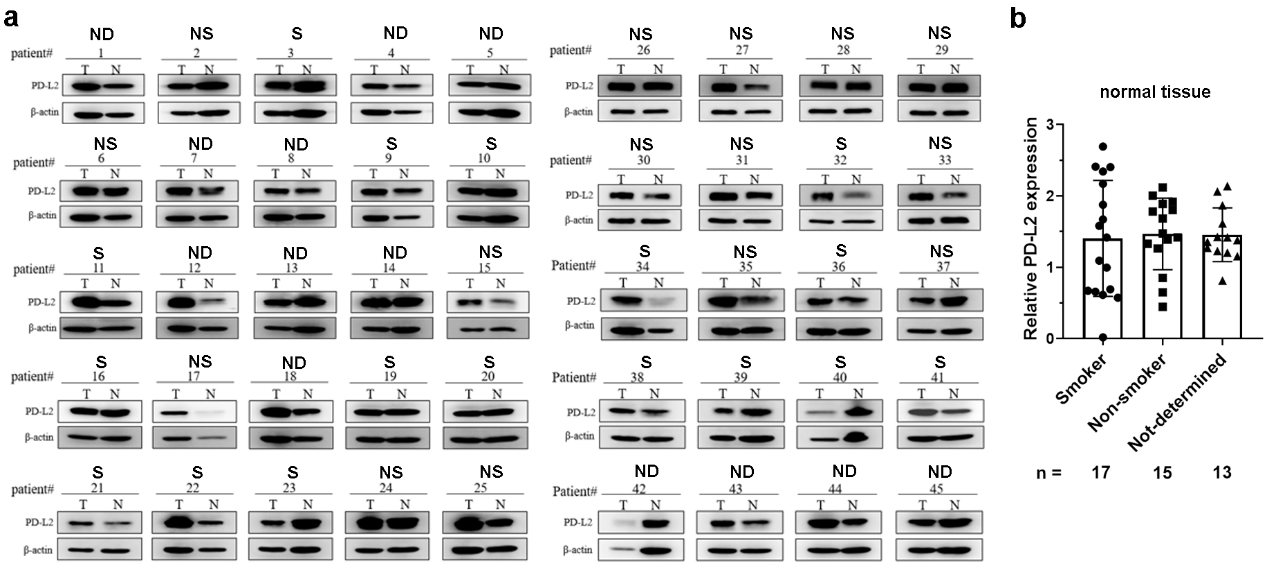


**Supplementary Figure 14.** **The expression of PD-L2 in human lung tumor (T) specimens and the adjacent normal (N) lung tissues was evaluated by western blot.** (**a**) all the western blot images for the 45 patients. ND: not-determined; NS: non-smoker; S: smoker. (**b**) Densitometry analysis of PD-L2 western blot bands normalized to β-actin in normal lung tissues.

**
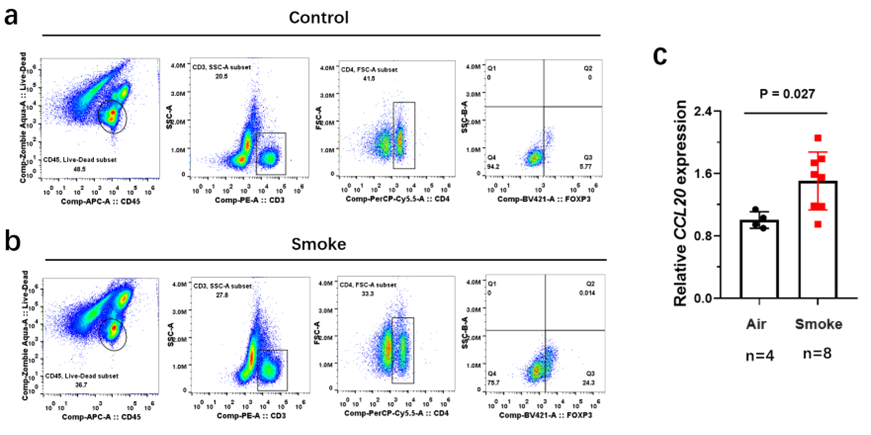
**

**Supplementary Figure 15. Flow cytometry analysis of the percentage of Tregs in the lung tissues of C57BL/6 mice (n = 8 per group) treated with fresh air or cigarette smoke for 60 days.** Male C57BL/6 mice were exposed to fresh air or cigarette smoke for 60 days, and then sacrificed with CO_2_ overdose. Lung tissues were collected for flow cytometry and qRT-PCR assays. Representative scatter plots of flow cytometry for (**a**) fresh air- and (**b**) cigarette smoke-treated mice. (**c**) The *CCL20* mRNA expression in the lung tissues of mice exposed to fresh air or cigarette smoke for 60 days. Student’s t test, Error bars, SD.


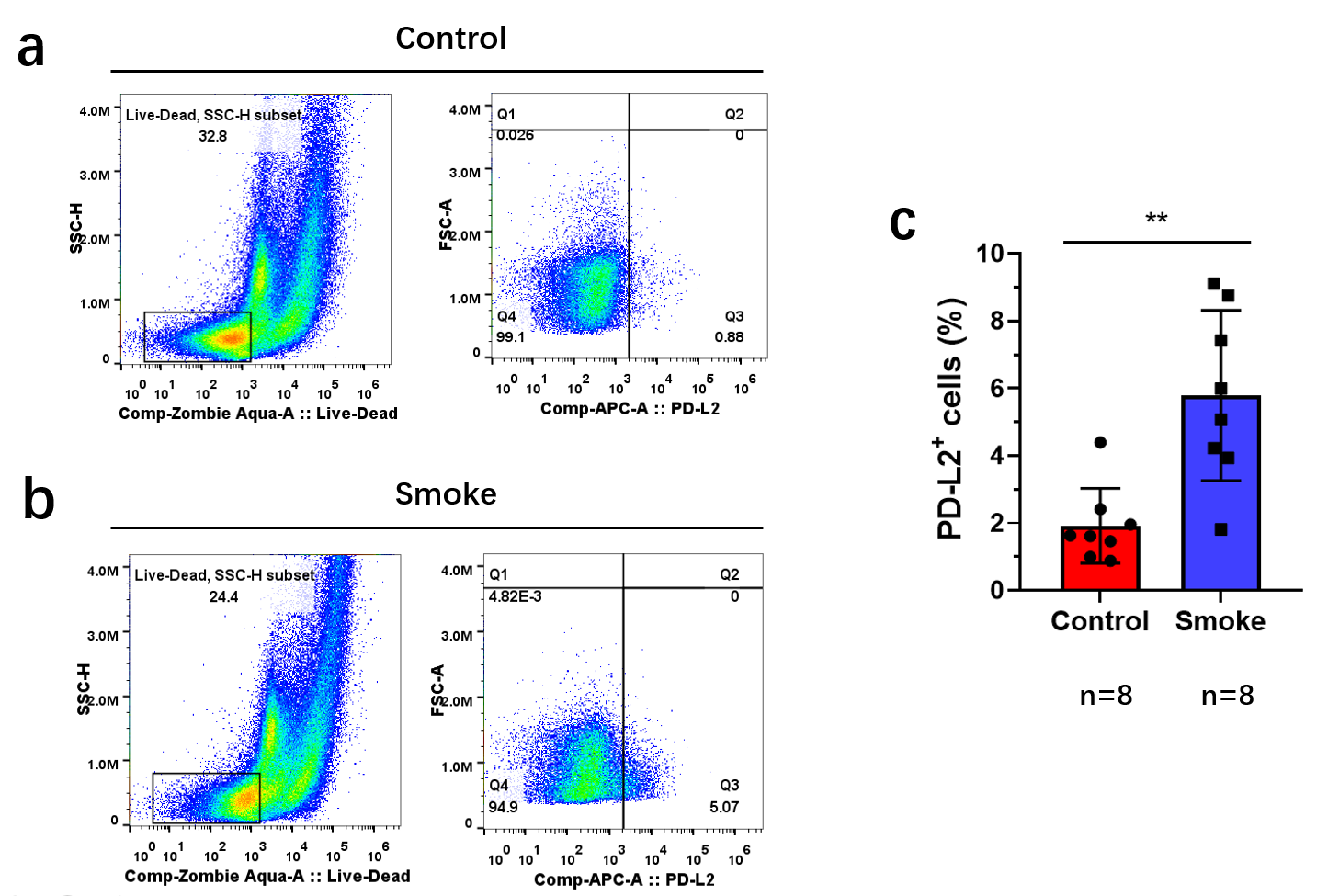


**Supplementary Figure 16. Flow cytometry analysis of PD-L2^+^ cells in the lung tissues of C57BL/6 mice (n = 8 per group) treated with fresh air or cigarette smoke for 60 days.** Male C57BL/6 mice were exposed to fresh air or cigarette smoke for 60 days, and then sacrificed with CO_2_ overdose. Lung tissues were collected for flow cytometry. Representative scatter plots of flow cytometry for (**a**) fresh air- and (**b**) cigarette smoke-treated mice. **c** Quantification of PD-L2^+^ cells in lung tissue of each group. Student’s t test, ** P < 0.01. Error bars, SD.


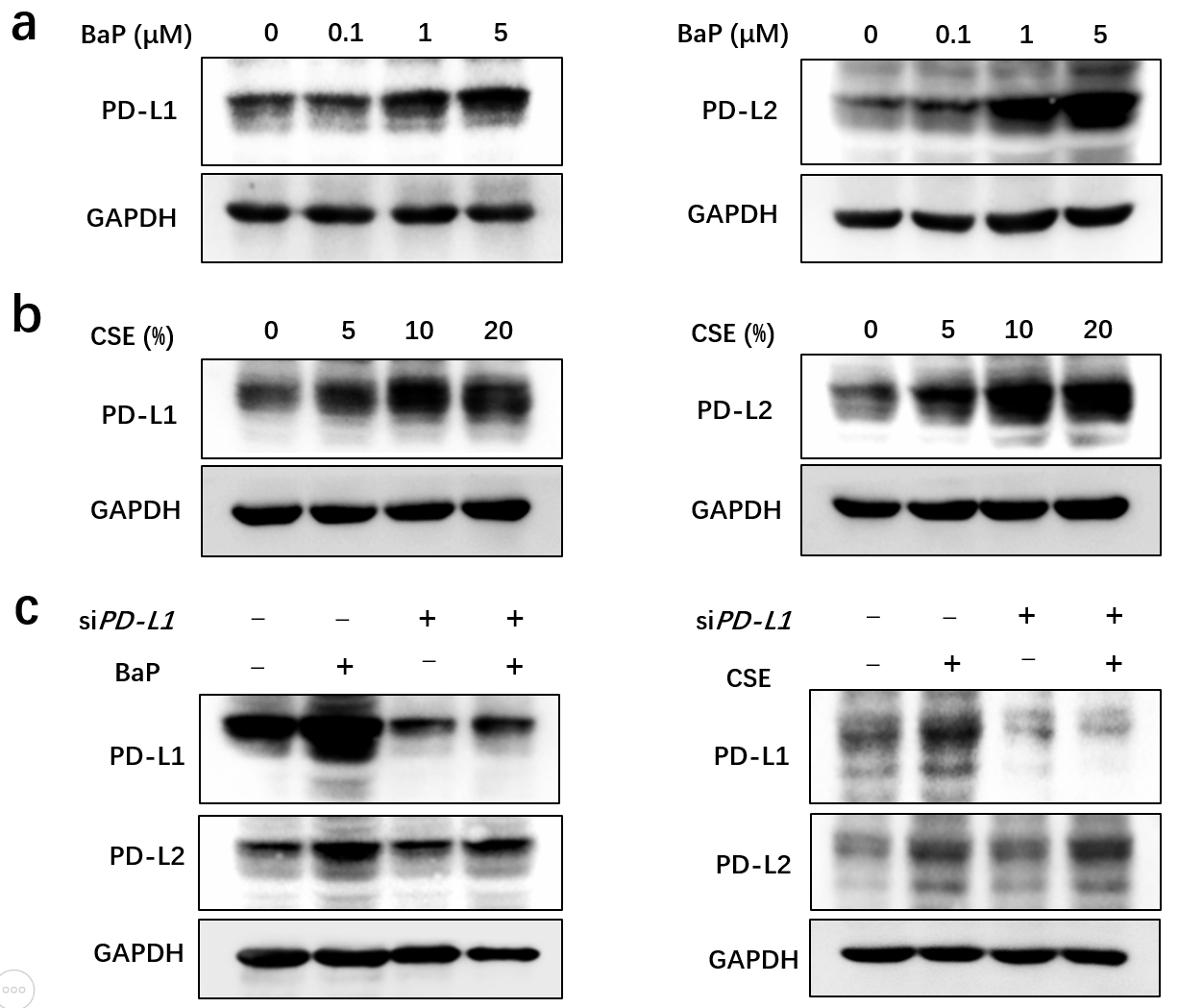


**Supplementary Figure 17. The expression of PD-L1 and PD-L2 in H460 cells treated with different concentration of BaP or CSE.** H460 cells were treated with (**a**) BaP or (**b**) CSE for 48 h, and then collected for Western blot analysis. (**c**) H460 cells were transfected with si*PD-L1* and co-treated with BaP (5 μM) or 10% of CSE for 48 h, and then collected for Western blot analysis.


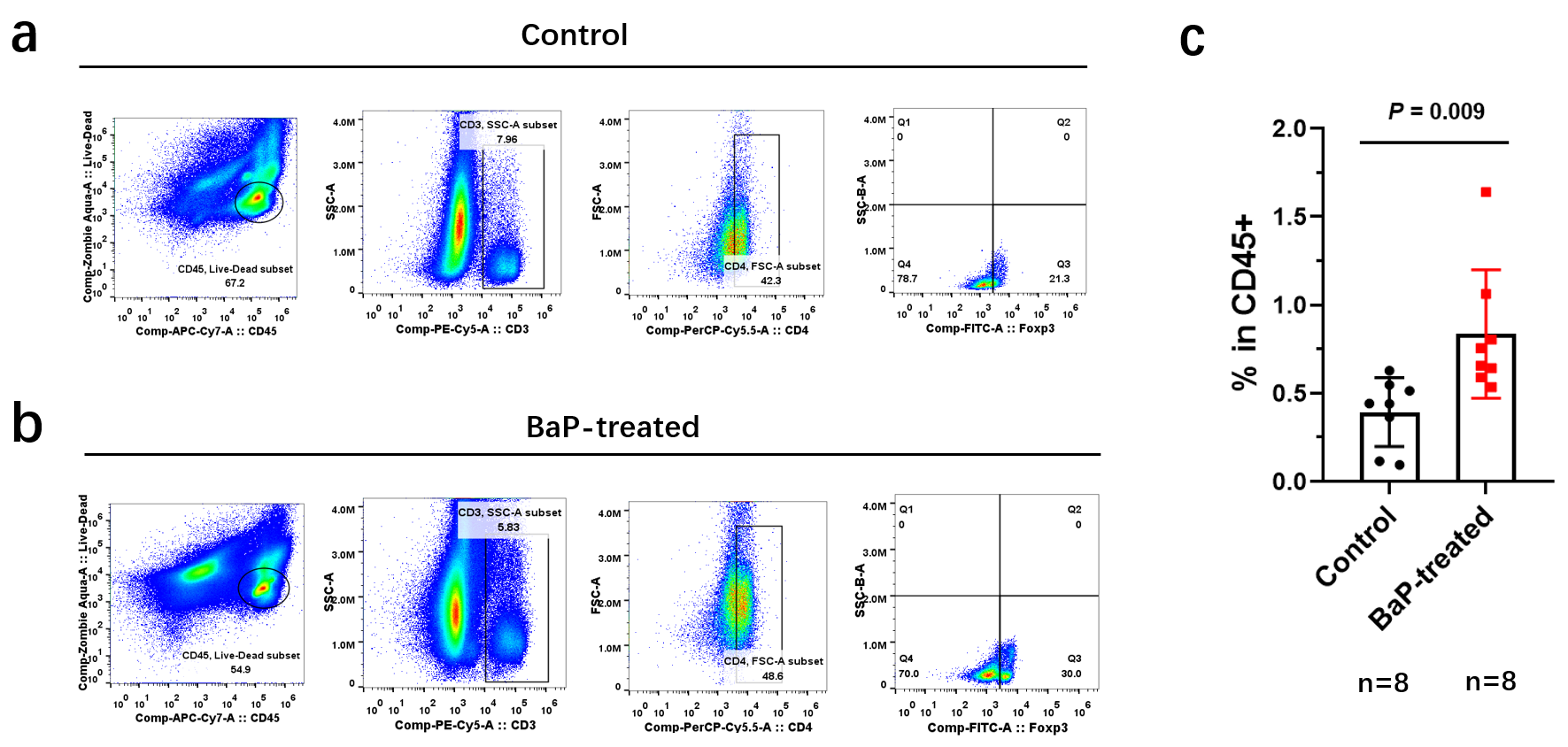


**Supplementary Figure 18. Flow cytometry analysis of the percentage of Tregs in the lung tissues of LLC-tumor bearing mice (n = 8 per group) treated or untreated with BaP for 3 weeks.** Female C57BL/6 mice were intravenously injected with 5 ×10^5^ of LLC cells, and then treated or untreated with 100 mg/kg BaP for 3 weeks. After that, mice were sacrificed with CO_2_ overdose. Lung tissues were collected for flow cytometry. Representative scatter plots of flow cytometry for (**a**) Bap-untreated and (**b**) -treated mice. **c** Quantification of FOXP3^+^ Treg cells in lung tissue of each group. Student’s t test. Error bars, SD.


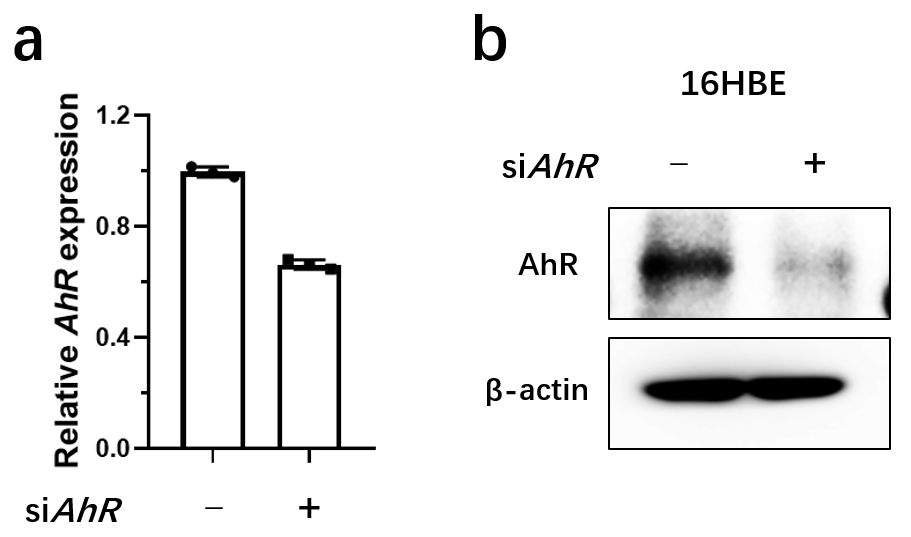


**Supplementary Figure 19. The AhR expression in 16HBE cells after si*AhR* transfection was measured by (a) qRT-PCR and (b) Western blot assays.**


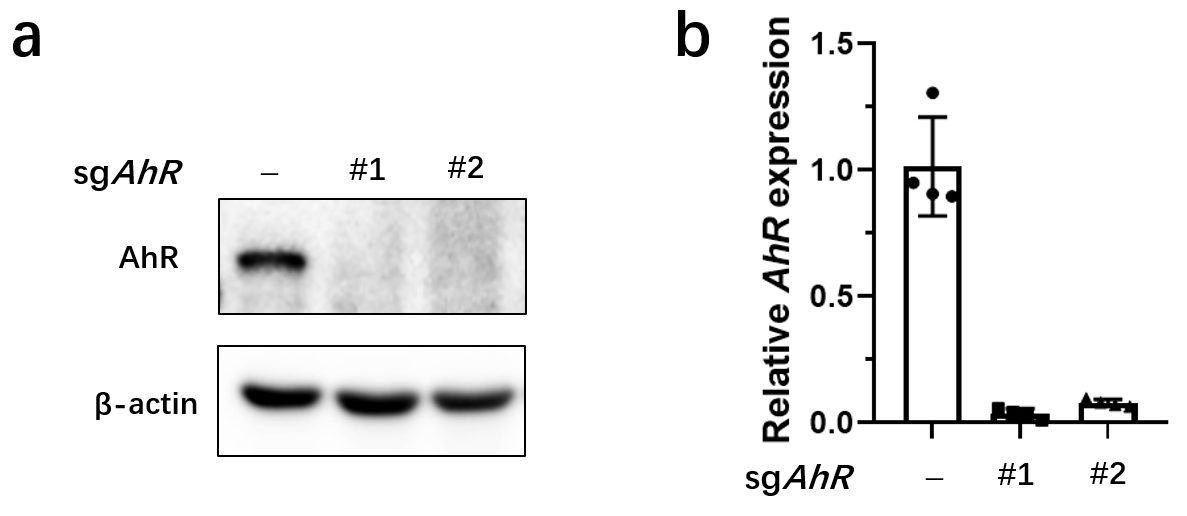


**Supplementary Figure 20. The AhR expression in H520 cells after knocking out AhR by (a) Western blot and (b) qRT-PCR assays.**


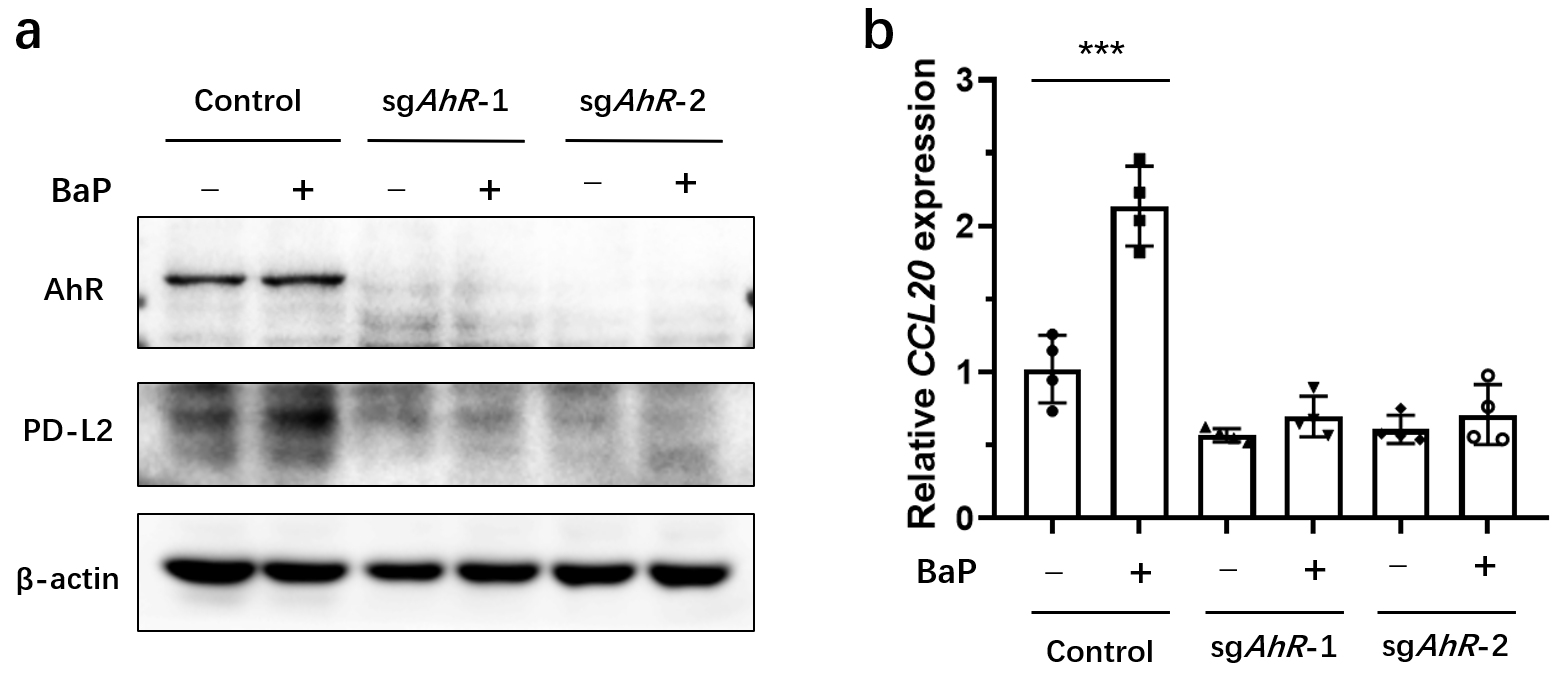


**Supplementary Figure 21. BaP induces PD-L2 and CCL20 expression in an AhR-dependent manner.** (**a**) PD-L2 protein expression and in AhR knockout cell line was measured by Western blot after treatment with 5 μM of BaP for 48 h. (**b**) *CCL20* mRNA expression in AhR knockout cell line was measured by RT-qPCR after treatment with BaP. Student’s t test, *** P < 0.001. Error bars, SD.


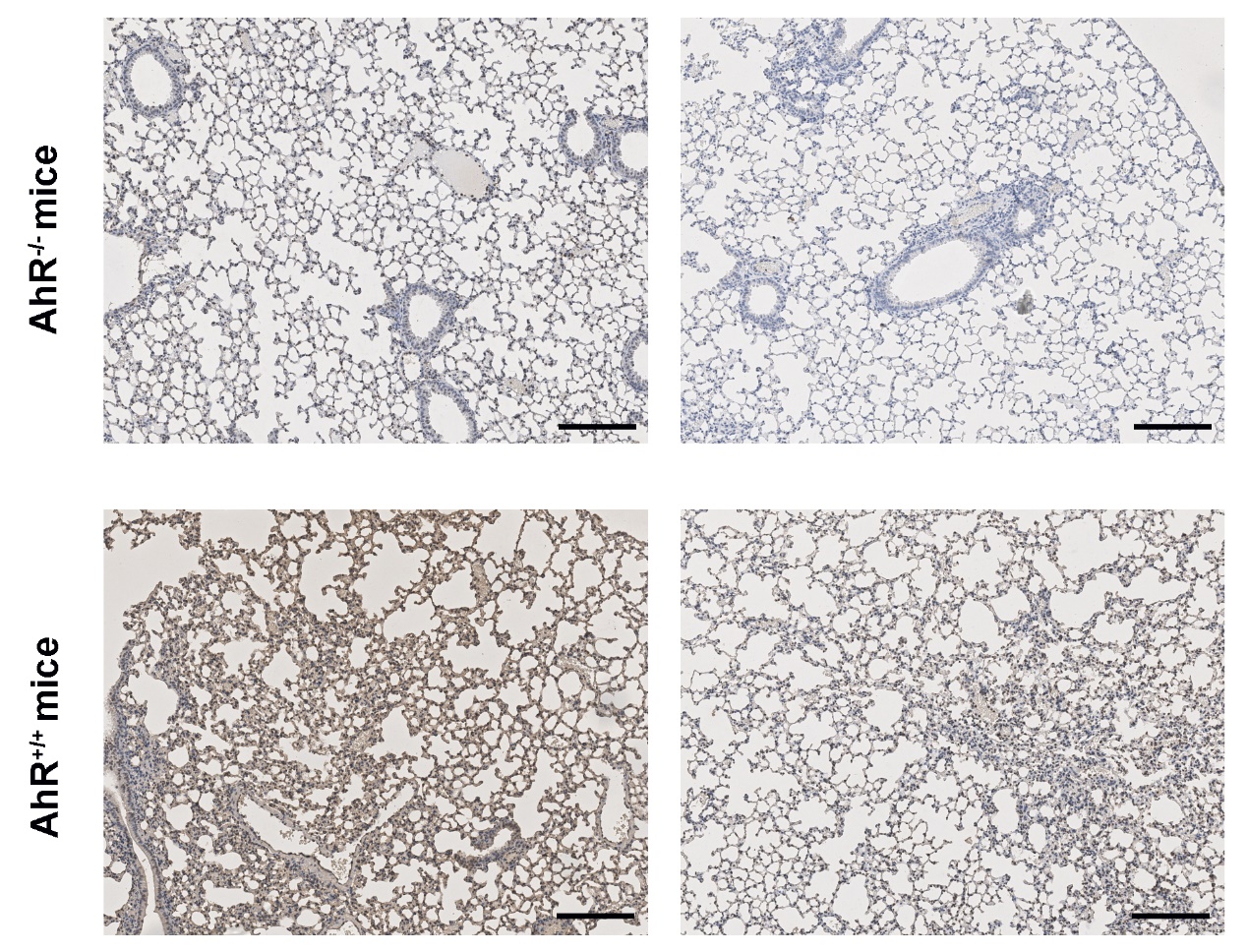


**Supplementary Figure 22. Representative IHC images of lung tissues from AhR^-/-^ and AhR^+/+^ mice after Foxp3 IHC staining.** Scale bars = 200 μm.

Table S1. Primers used in this study.

| qRT-PCR primers |  |
| --- | --- |
| Mouse Ccl1 forward | GGCTGCCGTGTGGATACAG |
| Mouse Ccl1 reverse | AGGTGATTTTGAACCCACGTTT |
| MouseCcl2 forward | TTAAAAACCTGGATCGGAACCAA |
| Mouse Ccl2 reverse | GCATTAGCTTCAGATTTACGGGT |
| Mouse Ccl3 forward | TTCTCTGTACCATGACACTCTGC |
| Mouse Ccl3 reverse | CGTGGAATCTTCCGGCTGTAG |
| Mouse Ccl4 forward | TTCCTGCTGTTTCTCTTACACCT |
| Mouse Ccl4 reverse | CTGTCTGCCTCTTTTGGTCAG |
| Mouse Ccl5 forward | GCTGCTTTGCCTACCTCTCC |
| Mouse Ccl5 reverse | TCGAGTGACAAACACGACTGC |
| Mouse Ccl7 forward | GCTGCTTTCAGCATCCAAGTG |
| Mouse Ccl7 reverse | CCAGGGACACCGACTACTG |
| Mouse Ccl8 forward | TCTACGCAGTGCTTCTTTGCC |
| Mouse Ccl8 reverse | AAGGGGGATCTTCAGCTTTAGTA |
| Mouse Ccl11 forward | GAATCACCAACAACAGATGCAC |
| Mouse Ccl11 reverse | ATCCTGGACCCACTTCTTCTT |
| Mouse Ccl17 forward | TACCATGAGGTCACTTCAGATGC |
| Mouse Ccl17 reverse | GCACTCTCGGCCTACATTGG |
| Mouse Ccl19 forward | GGGGTGCTAATGATGCGGAA |
| Mouse Ccl19 reverse | CCTTAGTGTGGTGAACACAACA |
| Mouse Ccl20 forward | ACTGTTGCCTCTCGTACATACA |
| Mouse Ccl20 reverse | GAGGAGGTTCACAGCCCTTTT |
| Mouse Ccl22 forward | AGGAGGATTGTCCACATGGAA |
| Mouse Ccl22 reverse | CTTGGCGTTCTAACCACCGA |
| Mouse Ccl24 forward | ATTCTGTGACCATCCCCTCAT |
| Mouse Ccl24 reverse | TGTATGTGCCTCTGAACCCAC |
| Mouse Ccl25 forward | TTACCAGCACAGGATCAAATGG |
| Mouse Ccl25 reverse | CGGAAGTAGAATCTCACAGCAC |
| Mouse Ccl26 forward | TTCTTCGATTTGGGTCTCCTTG |
| Mouse Ccl26 reverse | GTGCAGCTCTTGTCGGTGAA |
| Mouse Ccl28 forward | GTGTGTGGCTTTTCAAACCTCA |
| Mouse Ccl28 reverse | TGCATGAACTCACTCTTTCCAG |
| Mouse Cxcl1 forward | CTGGGATTCACCTCAAGAACATC |
| Mouse Cxcl1 reverse | CAGGGTCAAGGCAAGCCTC |
| Mouse Cxcl2 forward | CCAACCACCAGGCTACAGG |
| Mouse Cxcl2 reverse | GCGTCACACTCAAGCTCTG |
| Mouse Cxcl5 forward | GTTCCATCTCGCCATTCATGC |
| Mouse Cxcl5 reverse | GCGGCTATGACTGAGGAAGG |
| Mouse Cxcl7 forward | CTCAGACCTACATCGTCCTGC |
| Mouse Cxcl7 reverse | GTGGCTATCACTTCCACATCAG |
| Mouse Cxcl9 forward | TCCTTTTGGGCATCATCTTCC |
| Mouse Cxcl9 reverse | TTTGTAGTGGATCGTGCCTCG |
| Mouse Cxcl10 forward | CCAAGTGCTGCCGTCATTTTC |
| Mouse Cxcl10 reverse | GGCTCGCAGGGATGATTTCAA |
| Mouse Cxcl11 forward | GGCTTCCTTATGTTCAAACAGGG |
| Mouse Cxcl11 reverse | GCCGTTACTCGGGTAAATTACA |
| Mouse Cxcl12 forward | TGCATCAGTGACGGTAAACCA |
| Mouse Cxcl12 reverse | TTCTTCAGCCGTGCAACAATC |
| Mouse Cxcl13 forward | GGCCACGGTATTCTGGAAGC |
| Mouse Cxcl13 reverse | GGGCGTAACTTGAATCCGATCTA |
| Mouse Cxcl16 forward | CCTTGTCTCTTGCGTTCTTCC |
| Mouse Cxcl16 reverse | TCCAAAGTACCCTGCGGTATC |
| Mouse Cx3cl1 forward | ACGAAATGCGAAATCATGTGC |
| Mouse Cx3cl1 reverse | CTGTGTCGTCTCCAGGACAA |
| Mouse Foxp3 forward | CCCAGGAAAGACAGCAACCTT |
| Mouse Foxp3 reverse | TTCTCACAACCAGGCCACTTG |
| Mouse PD-L2 forward | GGAAGGGGGCAATGAGGAGA |
| Mouse PD-L2 reverse | AGCGTGGAAGAGAAGCGTAGG |
| Mouse Gapdh forward | AGGTCGGTGTGAACGGATTTG |
| Mouse Gapdh reverse | GGGGTCGTTGATGGCAACA |
| Human GAPDH forward | GAAGGTGAAGGTCGGAGTC |
| Human GAPDH reverse | GAAGATGGTGATGGGATTTC |
| Human CCL20 forward | GCAAGCAACTTTGACTGCTG |
| Human CCL20 reverse | ATTTGCGCACACAGACAACT |
| siRNA, shRNA, sgRNA primers |  |
| siAhR | UCACAAACUAAUUUAAUCC |
| siRgmb | GUACCAAGCUGUGACAGAUGA |
| siP65 | GGACAUAUGAGACCUUCAAdTdT |
| siPD-L1 | GCAGUGACCAUCAAGUCCU |
| ShPD-L2-1 | GGAGCAATAACAGCCAGTT |
| ShPD-L2-2 | GGACAGTACCAATGCATAA |
| ShRGMB-1 | CCATGATCCTTGCAACTAT |
| ShRGMB-2 | CCAGTGAAGGACATCTATT |
| SgAhR-1 | AGCGGCATAGAGACCGACTT |
| SgAhR-2 | GGATAACTGTAGAGCAGCAA |
| ChIP primers | |
| PD-L2 forward | TTCATGTCTACTGAGGACCTCA |
| PD-L2 reverse | AGGATACCATCCGTTTGATT |

Table S2. Summary of baseline demographic characteristics of 24 patients receiving Sintilimab treatment.

| **Number** | **Gender** | **Age** | **Smoke** | **Drinking** | **Pathology** | **Stage** | **Response** |
| --- | --- | --- | --- | --- | --- | --- | --- |
| 1 | male | 62 | yes | no | Adenocarcinoma | IV | CR |
| 2 | male | 73 | yes | no | Adenocarcinoma | Ⅳ | CR |
| 3 | female | 57 | no | no | Adenocarcinoma | Ⅳ | PD |
| 4 | male | 58 | yes | no | Adenocarcinoma | Ⅳ | PD |
| 5 | male | 58 | yes | no | Squamous-cell carcinoma | Ⅳ | PD |
| 6 | female | 62 | no | no | Adenocarcinoma | Ⅳ | PD |
| 7 | female | 65 | no | no | Adenocarcinoma | IV | PD |
| 8 | male | 65 | no | no | Squamous-cell carcinoma | IIIA | PD |
| 9 | female | 65 | no | no | Adenocarcinoma | IV | PD |
| 10 | male | 65 | yes | yes | Squamous-cell carcinoma | Ⅳ | PD |
| 11 | female | 73 | no | no | Adenocarcinoma | Ⅳ | PD |
| 12 | male | 84 | yes | no | Squamous-cell carcinoma | Ⅳ | PD |
| 13 | female | 62 | no | no | Adenocarcinoma | Ⅳ | PR |
| 14 | female | 68 | no | no | Adenocarcinoma | Ⅳ | PR |
| 15 | male | 71 | yes | no | Squamous-cell carcinoma | ⅠB | PR |
| 16 | male | 71 | yes | no | Squamous-cell carcinoma | ⅣB | PR |
| 17 | male | 59 | yes | no | Adenocarcinoma | ⅣA | SD |
| 18 | male | 62 | yes | yes | Squamous-cell carcinoma | Ⅳ | SD |
| 19 | female | 63 | no | no | Squamous-cell carcinoma | Ⅳ | SD |
| 20 | male | 65 | yes | yes | Adenocarcinoma | Ⅳ | SD |
| 21 | male | 66 | no | no | Squamous-cell carcinoma | Ⅳ | SD |
| 22 | male | 66 | no | no | Adenocarcinoma | Ⅳ | SD |
| 23 | male | 71 | yes | no | Endocrine cancer | Ⅳ | SD |
| 24 | male | 60 | yes | no | Small cell carcinoma | Ⅳ | CR |

CR, complete response; PR, partial response; SD, stable disease; PD, progression of disease.
